# Pixels2Pose: Super-resolution time-of-flight imaging for 3D pose estimation

**DOI:** 10.1126/sciadv.ade0123

**Published:** 2022-11-30

**Authors:** Alice Ruget, Max Tyler, Germán Mora Martín, Stirling Scholes, Feng Zhu, Istvan Gyongy, Brent Hearn, Steve McLaughlin, Abderrahim Halimi, Jonathan Leach

**Affiliations:** ^1^ School of Engineering and Physical Sciences, Heriot-Watt University, Edinburgh EH14 4AS, UK.; ^2^ School of Engineering, Institute for Integrated Micro and Nano Systems, University of Edinburgh, Edinburgh EH9 3FF, UK.; ^3^ Imaging Sub-group, STMicroelectronics, Edinburgh EH3 5DA, UK.

## Abstract

Single-photon–sensitive depth sensors are being increasingly used in next-generation electronics for human pose and gesture recognition. However, cost-effective sensors typically have a low spatial resolution, restricting their use to basic motion identification and simple object detection. Here, we perform a temporal to spatial mapping that drastically increases the resolution of a simple time-of-flight sensor, i.e., an initial resolution of 4 × 4 pixels to depth images of resolution 32 × 32 pixels. The output depth maps can then be used for accurate three-dimensional human pose estimation of multiple people. We develop a new explainable framework that provides intuition to how our network uses its input data and provides key information about the relevant parameters. Our work greatly expands the use cases of simple single-photon avalanche detector time-of-flight sensors and opens up promising possibilities for future super-resolution techniques applied to other types of sensors with similar data types, i.e., radar and sonar.

## Teaser

Pixels2Pose uses a simple time-of-flight sensor and a neural network to track multiple people’s poses in three dimensions.

## INTRODUCTION

Pose estimation is the process of locating the position of human body parts via analysis of images, videos, and sensor data. Accurate tracking of human anatomy is important in several areas, including activity recognition in gaming (*1*), gesture identification in consumer electronics (*2*), behavioral analysis in medical monitoring (*3*, *4*), as well as form and functional analysis in professional sports (*5*). Three-dimensional (3D) pose estimation from depth images or depth videos has been performed across many different domains: fall detection of elderly (*6*–*8*), medical diagnosis (*9*), assistance in physical therapy (*10*, *11*), monitoring of patient sleep (*12*), sport coaching (*13*), interaction with robots (*14*), and general action recognition (*15*–*17*). As the application areas for pose estimation span a wide range, so too does the technology used for it. For example, the most accurate pose estimation uses markers or multiple sensors that are tracked in three dimensions. Accurate 3D tracking can also be obtained using high-resolution depth images or triangulation from multiple linked intensity cameras.

While advanced technology is known to provide accurate pose estimation, it is also desirable to have accurate tracking from the simplest possible technology. Approaching the problem from this perspective opens up opportunities where cost, size, and weight are crucial considerations, e.g., the consumer electronics market, autonomous and self-driving vehicles, and airborne vehicles such as drones. Here, we show that a simple, small, and cost-effective time-of-flight (ToF) sensor with only 4 × 4 pixels contains sufficient data for 3D tracking of multiple human targets. The dimensions of the sensor are only 4.9 mm by 2.5 mm by 1.6 mm, and the cost is of order a few dollars. These sensors are becoming widely deployed in consumer electronics and integrated into larger-scale equipment as they have the advantage of very small size, low cost, and low power consumption. For example, these sensors are being increasingly used for basic gesture recognition and object detection tasks in products ranging from drones, phones, vacuum cleaners, cars, to automatic soap dispensers.

Very accurate pose estimation can be achieved by placing markers on the body. For example, inertial markers that record motion by combining data from different sensors such as accelerometers, gyroscopes, or magnetometers can recover accurate body poses (*18*, *19*) and can be used in combination with images (*20*, *21*). They have been developed, for example, for clinical applications (*22*) and for tracking posture during sport (*23*, *24*). Marker-based pose estimation gives the most accurate results, but these technologies are expensive and time-consuming to use, and the requirement to wear sensors means that they are not practical for general applications. Accurate poses can also be estimated using several linked cameras viewing a scene from different angles (*25*–*28*). Reflective markers placed on the body or face are also commonly used for animation and special effects in computer games or films (*29*). These approaches are very reliable, but it is desirable to have methods that do not require multiple cameras or use any markers.

3D pose estimation from single–point-of-view intensity images is an attractive alternative to labeled tracking because these images are easy to obtain (*5*, *30*). However, 3D pose estimation in this manner is extremely challenging because of depth ambiguities and occlusions from objects. Recent algorithms based on machine learning networks have achieved 3D pose estimation from single red-green-blue (RGB) images, demonstrating the reconstruction of multiple people that is robust to occlusions, in real time, and in both controlled and uncontrolled environments (*31*–*40*). The study by Ionescu *et al.* (*39*) provides a large database for 3D sensing of humans in natural environments, and the study by Zhe (*41*) contains a comprehensive collection of resources on pose estimation from RGB images.

An alternative to using RGB images is using depth images to reconstruct 3D poses (*42*–*44*). Depth images provide a considerable advantage because they already contain 3D information; however, more advanced sensors are required to record depth. For fast depth data acquisition, two main technologies are used: ToF cameras or structured light sensors. ToF cameras use a pulse of light to illuminate a target, and a detector records the returned light. They enable the acquisition of depth images at extremely low photon fluxes using first-photon events (*45*). Single-photon avalanche detector (SPAD) arrays are an emerging ToF technology for depth estimation. They enable acquisition of depth images at a high frame rate and they have a single-photon sensitivity, which enable to get images in degraded environments. However, the spatial resolution of this technology is typically low in comparison to the intensity images recorded by conventional cameras. Structured light sensors project a pattern of light onto the scene, and the depth measurement is based on triangulation (*46*). This has been used to reconstruct high-resolution depth images from a single-pixel detector at high frame rates (*47*) and from indirect light measurements of static scenes (*48*). However, the hardware requirements for structured illumination make it impossible at this stage to be integrated into high-scale marketed consumer devices. Many of these methods take inspiration from quantum imaging; see (*49*) for a comprehensive review of these computational imaging techniques.

An attractive solution to the requirement to use structured illumination for single-pixel depth imaging was proposed by Turpin *et al.* (*50*) who demonstrated that the rich temporal data from a single point in space contain information that could be converted to depth images via reconstruction with a neural network. This work shows an important proof of concept that good spatial resolution can be retrieved from temporal data rather than from the detector’s spatial structure. Estimating depth from a single pixel appeared to be a heavily ill-posed inverse problem, yet the authors show that the use of a static background overcomes this apparent constraint.

Computational imaging methods are known to provide powerful tools to extract and convert information between different modalities, provided that the input signal is rich and the task is sufficiently restricted. The work in reference (*50*) was developed further to show depth imaging of people using multipath temporal echoes from radar, sonar, and lidar data (*51*), and the poses of humans that are behind walls can be estimated using data obtained at radar frequencies (*52*, *53*). In addition, networks that use multiple input data sources have been used for data fusion to increase the resolution of depth images originating from the temporal histogram data from single-photon detector array sensors and intensity images (*54*–*57*).

On the other hand, small, cost-effective ToF depth detectors with very few pixels have been developed for commercial purposes and are designed for applications such as autofocus assist or obstacle detection in smartphones and drones. While they sensors only have a few pixels, the data that they record can have rich temporal information, and Callenberg *et al.* (*58*) recently demonstrated a range of applications that are significantly enhanced by use of the full ToF histogram data from a cheap commercial SPAD sensor. This work highlights the increasing range of applications that can be delivered from such a ToF sensor.

Our work builds upon the core ideas of image enhancement using neural networks and processing the full histogram data obtained from cost-effective ToF SPAD sensors. We show that generating depth images from a cheap, simple depth sensor can be achieved at high frame rates. Not only can we reconstruct depth images, but these images also have sufficient resolution to perform accurate 3D pose estimation of multiple targets. Crucially, as the sensor has multiple pixels, our system solves a more constrained ill-posed problem, and therefore, the pretrained network works in a range of different environments.

In addition, we provide an explainable framework that gives the intuition as to how the network interprets the input data. The use of the full ToF histogram data from a few pixels is key to the success of the networks, and the framework gives insights into how these data are used. The framework identifies the core parameters in the histogram dataset and, thus, provides valuable information for the development of future SPAD sensors or other sensors that provide similar data types, e.g., radar and sonar. Our results provide a pathway for devices to perform at much higher frame rates by establishing and then outputting only the core information from the sensor that is essential.

The novelty in our results lies in the fact that we are able to track multiple people in three dimensions using a sensor designed for much simpler tasks, and we are able to establish the core information contained in the input data. From first consideration of the resolution of the sensor (4 × 4 pixels), one would not think that this is possible. However, our method is able to reformat the rich information in the time domain to increase the resolution in the spatial domain without the requirement of a fixed background. The increased resolution in the spatial domain is sufficient to identify poses of multiple humans in three dimensions. Therefore, this work greatly expands the use cases of simple SPAD ToF sensors.

## RESULTS

### Overview of the system

**Fig. 1.**
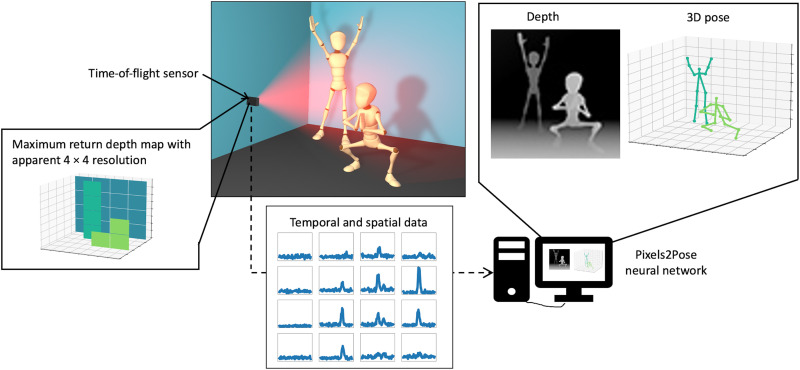
Schematic of the Pixels2Pose system. A small, cost-effective ToF sensor illuminates a scene and generates histogram data with a spatial resolution of 4 × 4 (*x*, *y*). These data are passed to the Pixels2Pose network to generate accurate pose reconstruction in 3D.

The Pixels2Pose system uses a small sensor to illuminate a scene and generate ToF histogram data of size 4 × 4 × 144 (*x*,*y*,*t*). These data are then passed to a neural network that has been trained to recover the poses of multiple people in three dimensions. The training stage of Pixels2Pose uses high-resolution depth and intensity images obtained from a Microsoft Kinect sensor and the RGB-based pose network OpenPose (*59*). Despite the apparent low spatial resolution, after the supervised training, our proposed Pixels2Pose system transforms the sensor’s rich ToF data into accurate 3D pose data of multiple people. A schematic of the system is shown in Fig. 1.

Our Pixels2Pose system is made of two neural networks: one that estimates depth from measured histograms and one inspired from the network OpenPose (*59*) that creates 2D poses using heatmaps of joints and part affinity fields. Our final step consists of superimposing the two outputs to render a 3D pose. We demonstrate continuous real-time video at a frame rate of 7 fps. Our approach can be adopted widely in a range of systems because of the simplicity of the underlying technology.

### Sensor

The key sensor for our work is the vl53l5 SPAD sensor manufactured by STMicroelectronics. The sensor illuminates the scene with 940-nm light pulses, and its SPAD detectors record the time of arrival of photons reflected as histograms of photon counts. The field of view is 60° diagonal, the maximum range is 3 m, and the frame rate is 10 fps. The dimensions of the sensor are 4.9 mm by 2.5 mm by 1.6 mm, the spatial resolution is only 4 × 4 pixels, and the temporal resolution is 144 time bins, each separated by 125 ps. The data are cropped to 100 time bins so that there are no unwanted artifacts from objects in the background. We can establish the main depth in each pixel, i.e., a single depth associated with the time bin showing maximum return of photons. This provides a 4 × 4 maximum return depth map. A visual representation of the temporal and spatial data from the vl53l5 sensor and its corresponding maximum return depth map are shown in Fig. 1.

### Pixels2Pose network

**Fig. 2.**
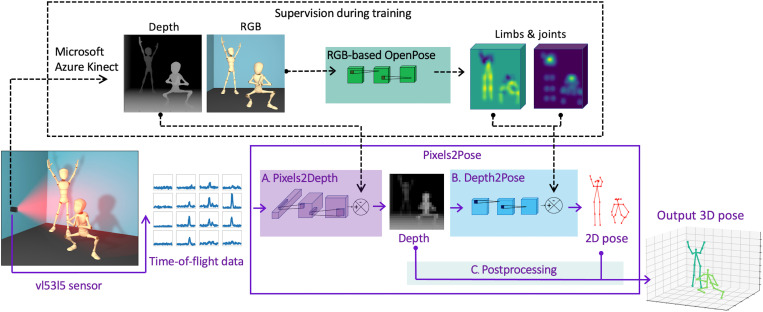
Overview of Pixels2Pose along with the supervision used for training. The bottom part displays the Pixels2Pose system. The ToF data of the sensor are passed through a series of three steps to reconstruct the 3D pose: (**A**) The network Pixels2Depth returns a high-resolution (HR) depth map from the histogram data; (**B**) the network Depth2Pose processes the HR depth map to return 2D poses; (**C**) the HR depth map and the 2D poses are combined to produce 3D poses. The top part displays the system used for the training of the networks Pixels2Depth and Depth2Pose. A Microsoft Azure Kinect DK camera is used to provide the labels corresponding to the sensor data. For Pixels2Depth, the HR depth images of the Kinect are used as labels. For Depth2Pose, the RGB image is processed through OpenPose (*59*) to get the 2D pose labels.

The proposed Pixels2Pose system takes the raw data of the sensor as its input, i.e., the 4 × 4 histograms of 100 time bins each, generates a higher-resolution depth map, and then uses the depth map to render the people poses in 3D. An overview of Pixels2Pose is displayed in Fig. 2. It consists of three steps: first, a neural network called Pixels2Depth; second, a neural network called Depth2Pose; and last, a postprocessing module that combines the information from each network. Pixels2Depth processes the histogram coming from the sensor using 3D convolutional layers to render depth maps with a resolution of 32 × 32 pixels. Depth2Pose then processes this higher-resolution depth map using 2D convolutional layers to output the 2D position of joints and limbs of all people present. This stage uses an adaptation of OpenPose (*59*) specifically written for depth images rather than intensity images. Last, we associate the limb locations provided by Depth2Pose with the corresponding depth locations obtained from Pixels2Depth to recover distinct 3D skeletons of people. Further details on the different steps are provided in section S2.

### Supervised training

The two networks that we use for Pixels2Pose, Pixels2Depth and Depth2Pose, are each trained separately and then combined later. To train Pixels2Depth, we simultaneously record histograms from the vl53l5 sensor and the corresponding depth images with a Microsoft Azure Kinect DK. The high-resolution images from the Kinect are down-sampled to 32 × 32 pixels using bicubic interpolation before training. We now have the data from the vl53l5 sensor and the corresponding ground truth depth label that can be used for training.

To train Depth2Pose, we exploit the corresponding Kinect’s RGB image, which is recorded at the same time as the depth image. We can use the intensity images to extract 2D pose labels (confidence maps of joints and limbs position) via the RGB-based model OpenPose (*59*). These 2D pose labels are the ground truth data used to train the Depth2Pose network. During training, Depth2Pose learns the parameters of the network to convert a depth image from Pixels2Depth to the 2D pose obtained from the RGB image. After the supervised training of the networks, Pixels2Pose relies only on the vl53l5 sensor data with no additional camera necessary.

Our proposed method takes the input from the vl53l5 sensor and first generates an up-scaled depth map. This depth map is then combined with the output of a modified version of the OpenPose network. In this manner, we are able to leverage the advantages of the OpenPose network, namely, that it is agnostic to the number of people in the scene, it deals with obscured body parts when people stand in front of one another, and it enables multiple people in the same scene to be tracked accurately. Tracking multiple people in the same scene is difficult as body parts have to be associated with the correct person, i.e., the hands and arms of a person should be attached to the torso of the same person. This association is achieved accurately by OpenPose. An alternative to our approach would have been end-to-end training, where the sensor data were transformed directly to 3D pose information. However, this approach would have required the association of body parts to the correct person to be implemented by our network.

Further to this, 3D convolution layers embedded within the Pixels2Depth network are used to best exploit the spatial and temporal information present in the data, while keeping the size of the network small. Solutions based on dense layers have been shown to convert temporal data to depth images (*50*), but these are typically larger networks with more parameters for the same level of performance. Future work here could also use recurrent networks to exploit the temporal correlations that are present from one frame to the next.

We trained three separate networks for reconstructing one, two, and three people in 3D. We collected 7000 images for the training for the one-person network, 9500 for the two-person network, and 9500 for the three-person network. All the training and validation images are captured in a controlled laboratory environment. Further details on the network structure and on the training are provided in section S2. To know which model to use (one, two, or three person), we could train an additional network to detect the number of people present in the scene from the sensor’s raw data.

### Pose estimation of multiple people in 3D

**Fig. 3.**
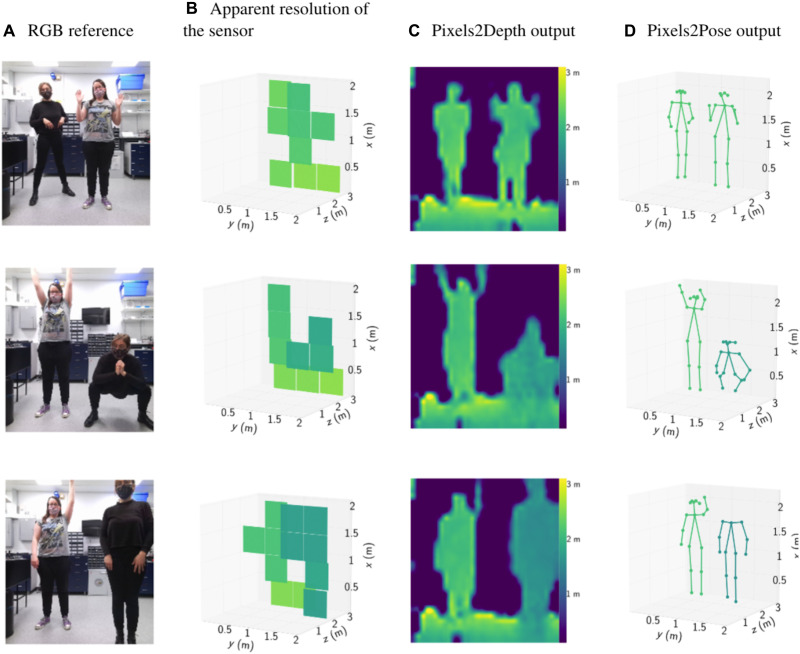
Validation results with two people. (**A**) The RGB image taken by a Kinect for reference. (**B**) The 4 × 4 depth map corresponding to the maximum return of photon counts of the 4 × 4 × 100 histogram. (**C**) The output of Pixels2Depth. (**D**) The reconstruction of Pixels2Pose. These results were generated using testing data that were not used in the training of the network.

**Fig. 4.**
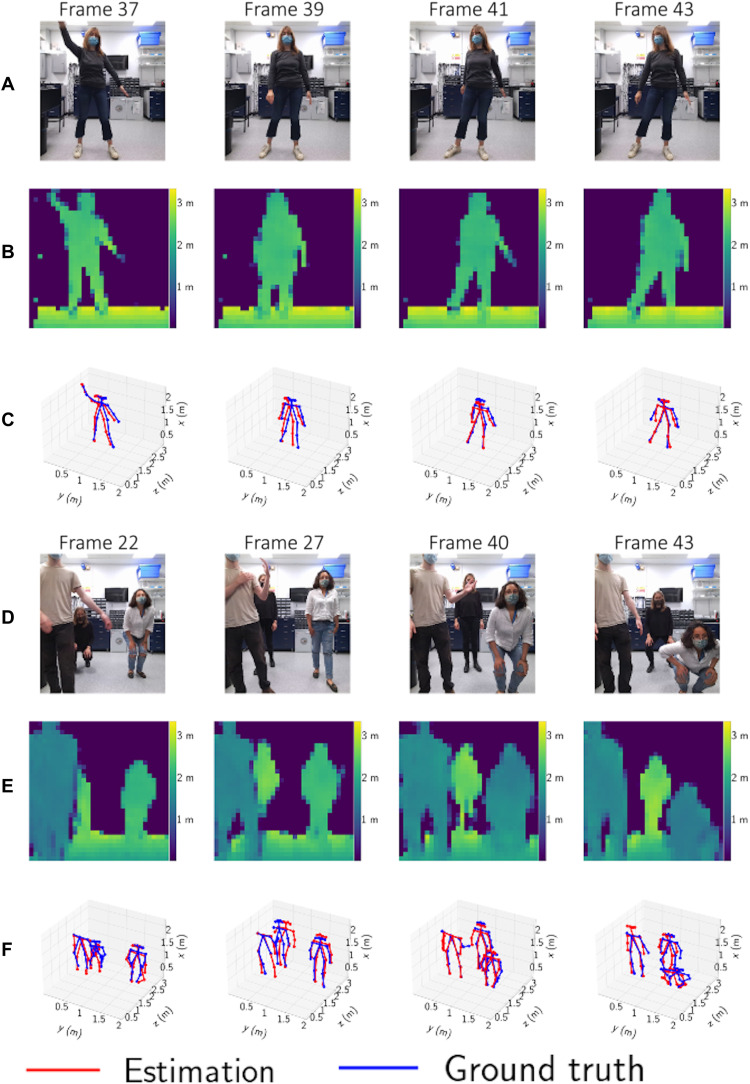
Validation results with one and three people. The depths reconstructions (**B** and **E**), 3D reconstructions (**C** and **F**), and the corresponding RGB reference images (**A** and **D**) are shown for different scenes containing one or three people. These results were generated using testing data that were not used in the training of the network.

Several frames showing the outputs of the two-person Pixels2Depth and Pixels2Pose networks are shown in Fig. 3. These results were generated using testing data that were not used in the training of the network. Figure 4 shows several test frames from the results for the one-person and three-person Pixels2Pose networks. Here, we also show the ground truth 3D pose as a reference for comparison. Note that for these results, the RGB images shown in Figs. 3 and 4 were not used in the networks and are shown as a guide for the reader (RGB images were used in the training but not testing of the networks).

The ground truth 3D poses are obtained directly from the intensity and depth images of the Kinect. We use the high-resolution RGB images and OpenPose (*59*) to calculate the ground truth 2D pose data. Each of the points in the 2D pose dataset corresponds to the *x*, *y* location of a joint. The *z* location of each of the data points is obtained by using the corresponding depth information obtained from the associated Kinect depth image.

Movies S1 to S3 show videos of data obtained from the Pixels2Depth and Pixels2Pose networks for one, two, and three people, respectively. We also show the input to the network, the vl53l5 sensor data, and the reference data obtained from the Kinect camera.

### Evaluation of performance

We evaluate the accuracy of the estimated 3D poses on a validation dataset of 1500 images. We use 500 frames for each scenario of one, two, and three people. In Table 1, we show the error in positions along the *x*, *y*, and *z* axes for each joint in every pose that we estimate. The error is defined as the root mean squared error (RMSE), expressed as (for the *x* axis)

$${\text{RMSE}}_{x}=\sqrt{\frac{1}{N}\sum_{i=1}^{N}{\left({\hat{x}}_{i}-x_{i}\right)}^{2}}$$ (1)

with *N* the number of validation frames, $\left(\hat{x},\hat{y},\hat{z}\right)$ the estimated positions, and (*x*, *y*, *z*) the ground truth positions. We report the average error AE, defined as

$$\text{AE}=\frac{1}{N}\sum_{i=1}^{N}\sqrt{{\left({\hat{x}}_{i}-x_{i}\right)}^{2}+{\left({\hat{y}}_{i}-y_{i}\right)}^{2}+{\left({\hat{z}}_{i}-z_{i}\right)}^{2}}$$ (2)

**Table 1.** Evaluation of the performance. We report the root mean squared error (RMSE) between the estimated and the ground truth position of each joint for each axis *x*, *y*, and *z*. We report the percentages of correct key points (PCK-15, PCK-20, and PCK-30), i.e., the ratio of estimated body parts for which the distance to the ground truth is below 15, 20, and 30 cm, respectively. We also report the percentage of detected parts.

	**RMSE_*x*/*y*/*z*_ (cm)**	**AE (cm)**	**PCK-15 (%)**	**PCK-20 (%)**	**PCK-30 (%)**	**% Detected**
Neck	5.4/6.0/8.5	9.5	80.0	88.0	92.0	100
Shoulders	5.8/12.4/9.2	12.3	72.5	80.2	86.3	99.7
Hips	4.4/8.8/9.1	10.2	77.8	83.3	91.6	99.5
Knees	5.6/11.1/10.1	11.9	72.1	81.7	89.8	98.1
Ankles	7.9/15.1/11.3	15.1	62.1	74.4	86.4	95.7
Elbows	17.7/19.9/13.4	19.6	60.9	68.6	75.4	94.3
Wrists	22.6/26/17.6	25.9	50	57.5	65.1	88.1

We also report the percentages of correct key points (PCK-15, PCK-20, and PCK-30), i.e., the ratio of estimated body parts for which the distance to the ground truth is below 15, 20, and 30 cm, respectively. We see that for the large core body parts, i.e., neck, shoulders, hips, and knees, more than 70% of the estimates are within 15 cm of the real position; for the smaller body parts at the extremities, i.e., ankle, wrists, and elbows, between 65 and 90% of estimates are within 30 cm. With regard to improving the accuracy and percentage of detected body parts, we believe that we could use the fact that we record at a high frame rate and use the similarities in one frame to the next. Here, one could use recurrent neural networks that are known to make use of temporal correlations in time sequences.

Movies S4 to S6 show the reconstruction of poses from Pixels2Pose, along with the ground truth obtained from the Kinect sensor. We see that the overall movement of the people is accurately recovered. Examples of most common failure cases of Pixels2Pose are shown in Supplementary Text. The network occasionally fails to identify arm movements when multiple people are present in the scene, movements over multiple time frames are sometimes unrealistic, and we also observe that people can disappear from the frame when crossing behind one another. In the Supplementary Materials, we evaluate the performance of the Pixels2Depth network, evaluating the quality of the depth images in terms of RMSE, the mean absolute difference (MAD), and delta metrics.

The model Pixels2Depth consists of 368,929 parameters of type float32 and requires about 4.7 MB of memory. The model Depth2Pose consists of 2,517,768 parameters of type float32 and 30 MB. For one frame, the processing time is 0.032 s for Pixels2Depth, 0.032 s for Depth2Pose, and 0.07 s for the postprocessing module, i.e., the total processing time of Pixels2Pose equates to around 7 to 8 fps, processed on an NVidia Tesla RTX 6000 graphical processing unit (GPU).

We can reduce the memory requirements of the networks using the Tensorflow Lite converter. This can be used to create an appropriately sized network for implementation on computing systems with less resource than a GPU, e.g., mobile and Internet of Things devices. Tensorflow Lite applies a post-training quantization to the trainable weights from floating-point to integer. After the conversion, the entire Pixels2Pose system requires only 5 MB of memory. We find that the reduced-size networks have a very similar performance to the original models, often performing to within a few percent of the main network. The exact details of the performance of the lite version of Pixels2Pose can be found in section S3. The lite models can be used directly on a Raspberry Pi 4, in real time together with the acquisition of the data. In this case, we can achieve a frame rate of 1 fps for both the acquisition and the processing of the data.

### Performance in other environments

**Fig. 5.**
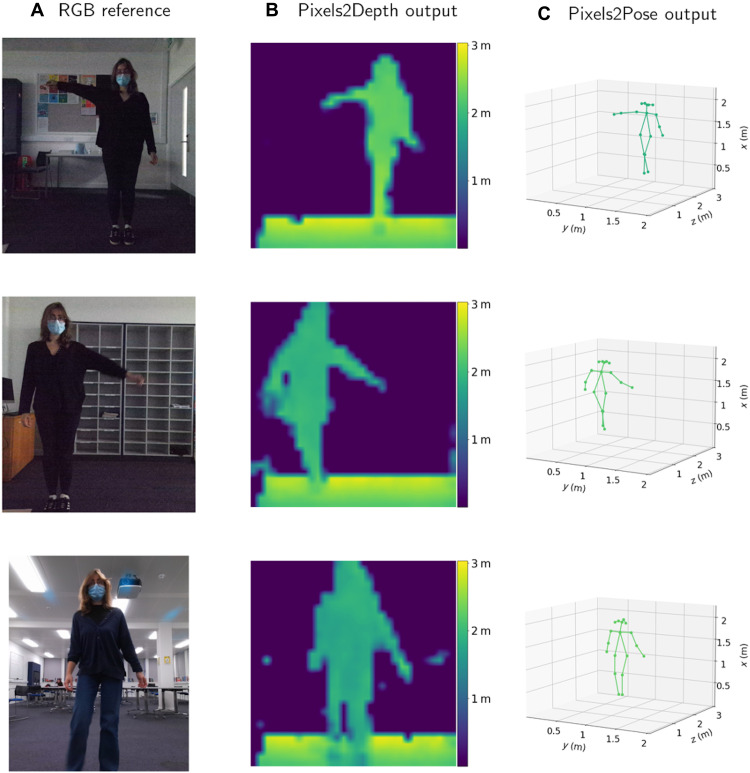
Results in other environments not used for training. We test the Pixels2Pose system in other rooms than the one used to record the training dataset. (**A**) The RGB image taken by a Kinect for reference. (**B**) The output of Pixels2Depth. (**C**) The reconstruction of Pixels2Pose. These results were generated using testing data that were not used in the training of the network.

To demonstrate that the trained Pixels2Pose network is transferable between different environments, we took new data with the vl53l5 sensor in a new room and from two different angles. No data from the second room were used in the training of the Pixels2Pose network. The acquired data were processed and 3D poses were reconstructed. Results are shown in Fig. 5. A video of the reconstruction in new environments is shown in movie S7.

As with the training data captured from the vl53l5, the number of bins from the histogram was reduced from 144 to 100. This ensures that there are no artifacts in the background that would affect the final result. We evaluate the accuracy of the estimated 3D poses on 500 images taken in two different environments. We report in table S4 the error in position in *x*, *y*, and *z* and the percentages of correct key points (PCK-15, PCK-20, and PCK-30), i.e., the ratio of estimated body parts for which the distance to the ground truth is below 15, 20, and 30 cm, respectively. The data show that the Pixels2Pose network recovers the 3D pose in an environment in which it was not trained, thus demonstrating the versatility of our system. We note that, in this case, the average error of the body locations increases, and this is likely due to changes in the ambient light levels and the precise orientation and location of the vl53l5 sensor with respect to the subject. These differences could be accounted for with further training of the network or a preprocessing step that corrects for orientation.

Mapping single-point temporal histogram data to spatial images is a heavily ill-posed problem (*50*). This is because there are many spatial positions in the scene that can lead to the same signal in a temporal bin at the detector. Inverting the temporal data therefore requires additional information. Turpin *et al.* solved this problem by using an asymmetric static background. In our case, we consider a sensor array that contains a few pixels in both vertical and horizontal dimensions, hence bringing limited but essential spatial information. For estimating pose, the stringent requirements on a fixed background are thus removed, and Pixels2Pose is transferable to different environments.

The system performs well in indoor environments because the ambient light levels recorded by the vl5315 sensor are low enough so as not to dominate the return signal from the laser. When the sensor is used outdoors in high ambient light levels, the SPAD pixels can saturate as the background illumination is too high. This type of performance is well known for SPAD sensors and is one of the limiting factors determining their use. The problem can be solved with the use of a higher power laser, but in turn, this causes laser eye-safety issues. Next-generation SPAD sensors, such as the one reported in (*60*), are designed to perform well under high–ambient light conditions, but further processing of the current sensor data would be required to extract the signal over the background in this situation.

In addition, we also limit our scenes to contain a maximum of three people in the field of view. This ensures that we maintain high performance for each of the situations that we consider. The extension of this work to perform well under a wider variety of environments, such as those reported in (*39*) and (*40*) is of great interest and will be the focus of future work.

### Explanation framework

Our system Pixels2Pose shows good performance on validation datasets acquired in different environments. However, it is essential to understand how the network interprets the input data to gain confidence and trust in its operation. An explainable artificial intelligence (XAI) technique called LIME (local interpretable model-agnostic explanations) has been extensively used to assess trust in machine learning models (*61*). The LIME framework seeks to explain models locally by learning an interpretable model around a prediction.

LIME was initially developed for classification problems, where the significance of a set of multidimensional input parameters are assessed with regard to a single classification output. In our case, however, the standard framework is not applicable because of the high dimensionality of both our histogram input and output depth images and poses. If we consider the Pixels2Depth network, for example, this takes an input raw histogram of size 4 × 4 × 100 and returns a super-resolved depth map of size 32 × 32. We have therefore developed a new framework to analyze the proposed network based on the input histogram data and output super-resolution depth map. This method analyses our models to provide intuition on how relevant features of the histogram data affect both the super-resolution task and the pose estimation.

We first consider Pixels2Depth for single-person reconstruction. We simplify the histogram input to a set of 48 Gaussian fit parameters, i.e., three parameters (position, amplitude, and width) for each of the 4 × 4 SPAD pixels. We then establish the impact of each of these parameters on every one of the 32 × 32 pixels in the final super-resolved depth image. This is achieved by creating a local model where the 48 input parameters are combined in a linear manner to provide every pixel in particular instance of a depth map (32 × 32 pixels). The weights of the linear model are influence maps, where the impact of each of the input parameters can be visualized. Please see the Supplementary Materials for the technical details and individual influence maps.

**Fig. 6.**
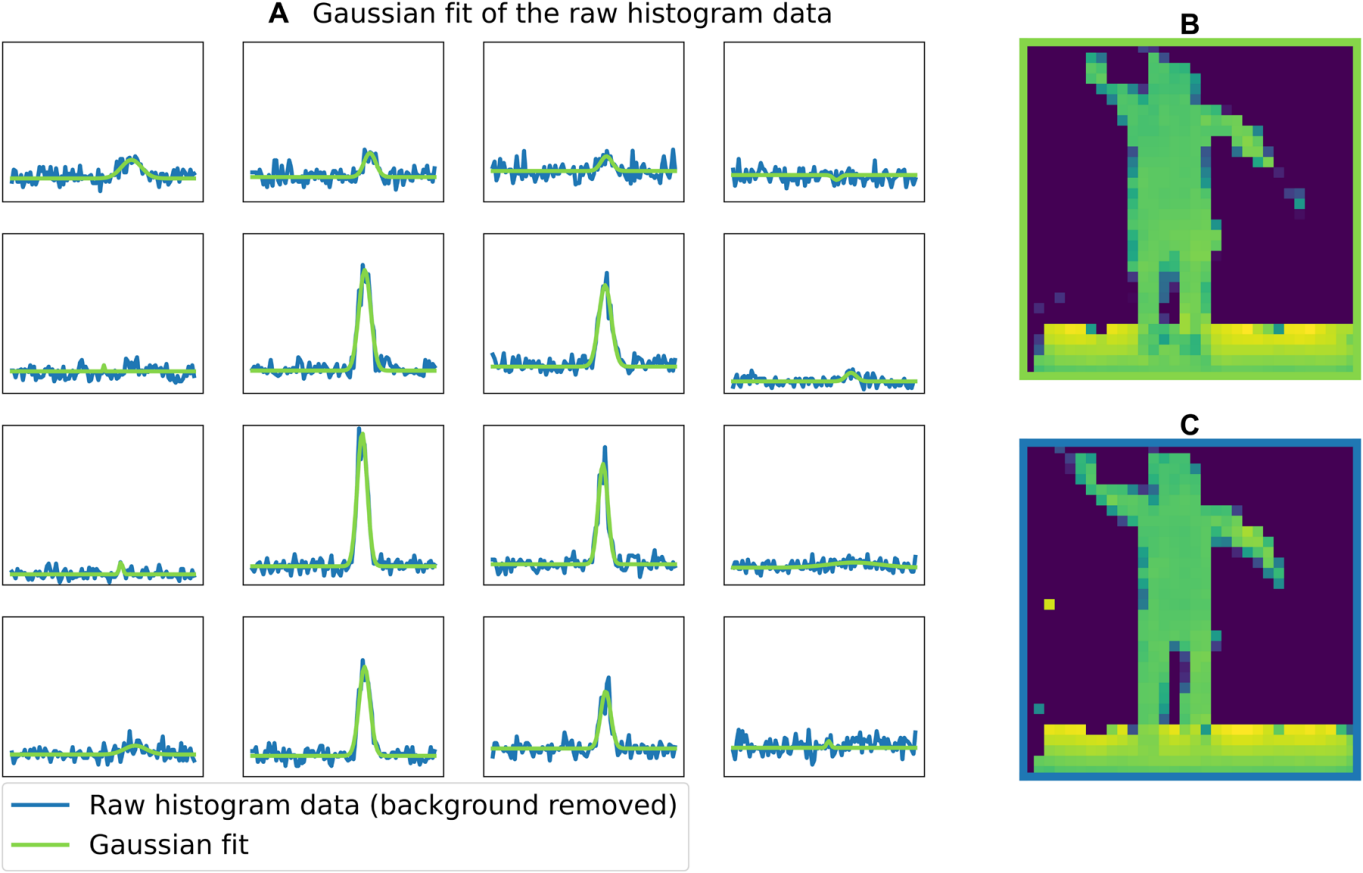
Gaussian fit of the histograms in the one-person scenario. (**A**) The raw histogram (blue) and the Gaussian fit (green). (**B**) The reconstruction by the network using the Gaussian fit data and (**C**) the reconstruction with the raw histogram data.

**Fig. 7.**
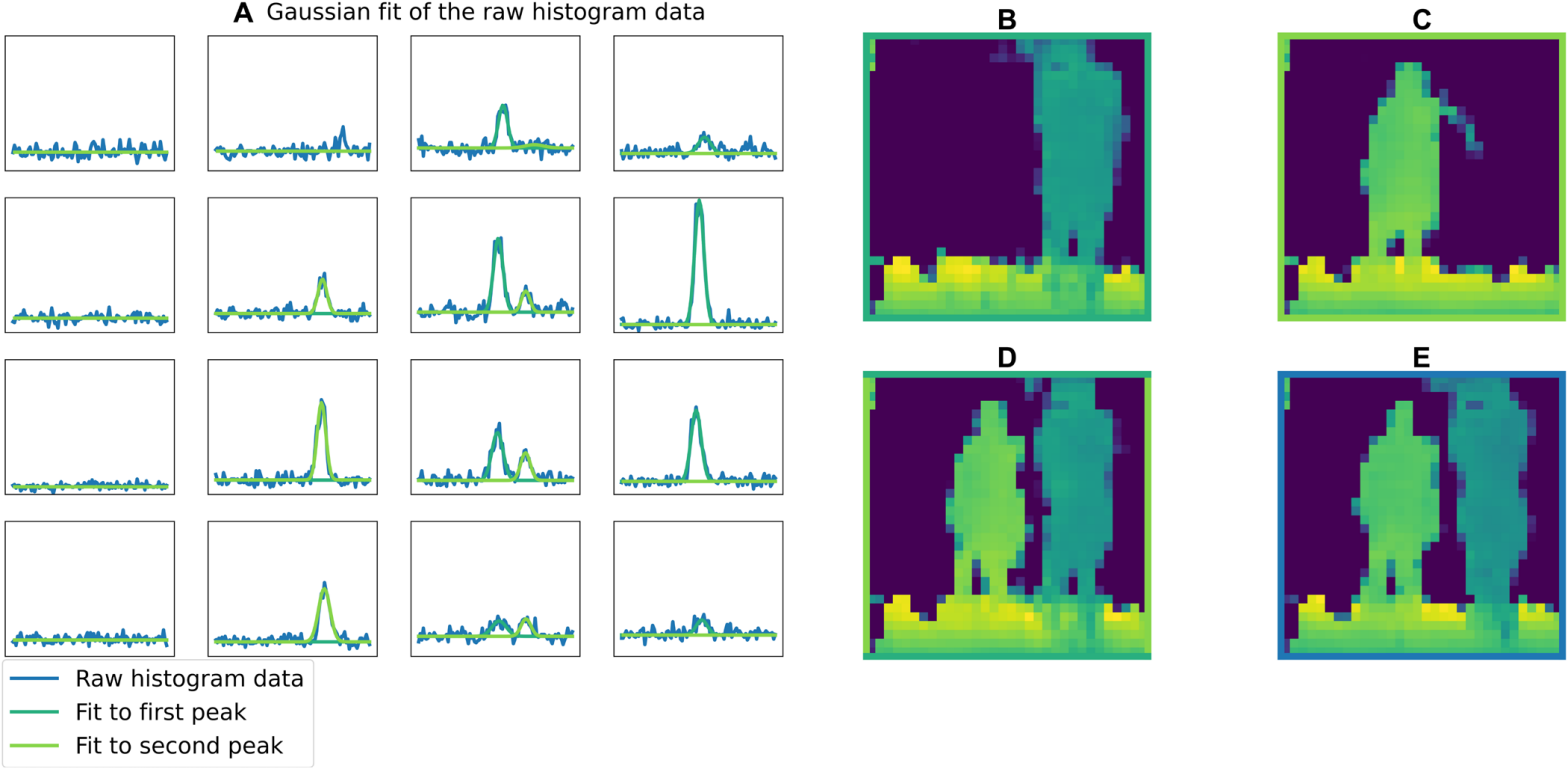
Gaussian fit of the histograms in the two-people scenario. (**A**) The raw histogram (blue), the first peak of the Gaussian fit (dark green), and second peak of the Gaussian fit (light green). (**B** and **C**) The reconstruction by the network obtained if only the first and the second peak are used. (**D**) The reconstruction if both peaks of the Gaussian fit are used. (**E**) The prediction from the raw histogram.

The first step is to find a more intuitive and simpler representation of the histogram data that can easily be analyzed. For that, we fit a Gaussian model to the histograms of each of the pixels. In the one-person scenario, we fit one Gaussian per pixel (see Fig. 6). Therefore, each histogram of 100 bins is condensed in four parameters: the amplitude, the width, the position of the peak, and the background level (the background level is not used in the local model). In the two-people scenario, two Gaussians per pixel are fitted, adding three extra parameters (see Fig. 7). Pixels2Depth reconstructs a very similar depth map from the Gaussian fitted histograms than from the initial raw histogram data (see Figs. 6 and 7). This result shows that Gaussians are a good proxy for the input histogram data, and the network could be reformatted to use these parameters (amplitude, position, and width) to reconstruct the depth map.

The second step is to fit a linear model to a local instance of the network. This model provides the intuition behind the relationship between the input data and the output depth maps. It reveals the importance/influence of the amplitude, width, and position of the fitted Gaussian peaks in the initial data on the final outcome. The proposed network of Pixels2Pose is a complex nonlinear model that is not easily interpretable. As in the LIME framework, we adopt a local linear approximation of the network, by focusing our analysis on an individual prediction of the Pixels2Depth network from the parameters of a single Gaussian fit (i.e., a single instance of input data). Using this linear approximation, we look at the importance of the amplitude, width, and position of the peaks on the final output. We choose the frames displayed in Figs. 6 and 7 as instances of interest for the one-person and two-people scenarios.

As Gaussians fitted to the histograms of the sensor’s data provide good depth maps when passed through the network, we seek to find a model that produces a similar depth map to Pixels2Depth but uses only a linear combination of the fit parameters. That is to say, each pixel in the final image is treated as a linear combination of the 48 fit parameters. It is necessary to create a new dataset for the local model around the prediction of interest. Here, we generate a set of 2000 new input parameters, giving the matrix *x* ∈ ℝ^2000×48^, where the amplitude, width, and position of the peaks for a random combination of pixels are changed by up to 10%. Each new set of 48 parameters is used to generate new data (dimension of 4 × 4 × 100) that is passed through the network, providing new depth images each of resolution 32 × 32. These 2000 new depth images are then are reformatted, providing the matrix *d* ∈ ℝ^2000×1024^. We then find the solution to the full linear model *xA* = *d*. We ensure sparsity in *A* by using weighted Lasso regression, where the distance between the original set of parameters and the new set of parameters is used. More details about the design of the linear model can be found in the Supplementary Materials. This solution provides the matrix *A* ∈ ℝ^48>×1024^ that gives the details about how the parameters in *x* are combined to generate the depth images *d*. The coefficients of the solution *A* are the weights applied to the appropriate parameters in *x*. Each of the 48 columns of *A* are of length 1024 and can be reshaped to an image of size 32 × 32. Each image is then associated with a single parameter from a single pixel and gives the influence of that parameter on the final image, where we define the influence as the absolute value of the weights. The influence of the amplitude, position, or width of all the 4 × 4 pixels in the input can be obtained by summing the appropriate influence maps from *A*

**Fig. 8.**
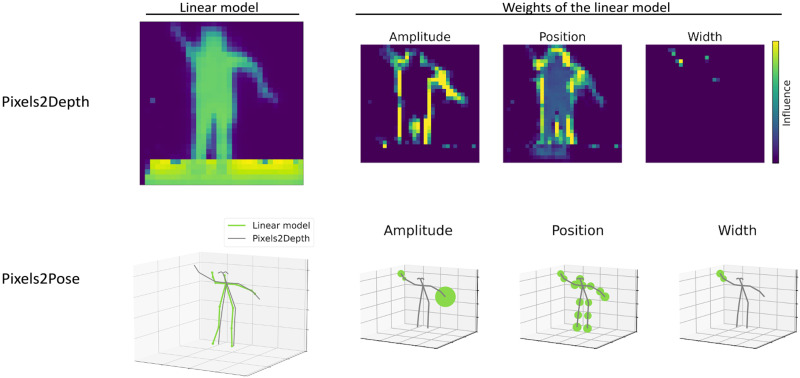
Explanation models for Pixels2Depth and Pixels2Pose in the one-person scenario. The first column shows the reconstruction of the instance of interest by the local explanation models for Pixels2Depth and Pixels2Pose. The last three columns show the combined influence of all initial 16 pixels on the depth maps and pose reconstruction. We display the sum of the absolute values of all the weights of the linear model for the different fit parameters. We see that the peak positions affect the depth of the full body and the shape of the edges, while the amplitude affects only the edges. For the pose estimation, the radius of the spheres is proportional to the weights of the linear model.

Figure 8 shows data for the one-person case and provides the reconstruction from the linear model and the corresponding influence maps associated to each set of Gaussian parameters. The results reveal that the influence of the amplitudes of the peaks is located only at the edges of the body, whereas the influence of the positions of the peaks is located at both the edge and central core of the body. We conclude, therefore, that the amplitudes and positions of the data establish the shape of the body in *x* and *y*, and the positions of the peaks establish the overall position of the person in the depth direction *z*. We note that the 10% variations in the width parameter do not have much impact on the reconstruction.

**Fig. 9.**
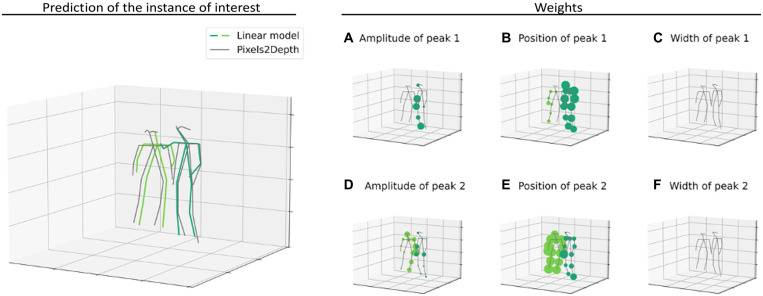
Explanation model for Pixels2Pose in the two-people scenario. On the left, we show the prediction of the instance of interest made by the network and made by the linear model. On the right, we show the combined influence of all initial 16 pixels on the pose reconstruction. The first row (**A** to **C**) shows the weights applied to the parameters of the first peak of the Gaussian fit, and the second row (**D** to **F**) shows the weights applied to the second peak of the Gaussian fit. The radius of the spheres is proportional to the weights of the linear model.

We apply the same strategy to build a local explanation model to the end-to-end system Pixels2Pose. In that case, we train a linear model to render the 3D position of the body parts from the parameters of the Gaussian fit. We fit the model around the instances of interest using the same dataset as previously. Figures 8 and 9 show the learnt weights applied to the amplitude, position, and width of the peak on the reconstruction of the different body parts for the one-person and two-people cases. In Fig. 8, we add together the 16 individual influence maps from each of the pixels to see the combined impact. These individual maps are shown in the Supplementary Materials.

In the one-person case (Fig. 8), only the extremities of the arms are linked to the amplitudes of the peak. In this frame of interest, the arms are in a special position compared to the rest of the body. We draw similar conclusions as for the depth reconstruction, i.e., the amplitudes of the peak have an influence on the finer details of the reconstruction and give the shape of the body. The position of the peaks has an impact on all of the body parts, and the width has less impact.

In the two-people case (Fig. 9), we distinguish the influence of the parameters of the first peak (first row) and of the second peak (second row). The weights applied to the parameters of the first peak affect mostly the person that is in the front of the image and vice versa. Again, the amplitudes of the peaks have an impact at the edges between the two persons. This could be explained by the fact that the ratio between the amplitudes of the first and second peaks is linked to the overlap between the two people. The position of one peak affects all the body parts of its associated person, and the width has no impact.

To summarize, here is what we conclude from the explainable framework: First, Gaussians with amplitudes, positions, and widths are a good fit to the data, and these are an appropriate proxy input to the model; second, if two people are present in the scene, then each person can be associated with separate Gaussian fits; and last, the local linear model reveals the influence of each of the sets of parameters of the fit to the final output. We also see that the critical parameters are the amplitude and positions of the peaks and not the widths. Once these parameters have been established from the initial histogram, we have collected all the necessary information for the super-resolution and pose estimation tasks. Operating in this fashion drastically reduces the number of parameters that a network would need to operate with.

Moving forward and applying our techniques to new systems (lidar, radar, and sonar), the precise benefit of having access to the full dataset (histograms) versus the key parameters (amplitudes and positions of peaks) and is yet to be established. It is already known that intensity event cameras, which only report the pixels in a scene that change from frame to frame, enable much higher frame rates and dynamic ranges. Applying this approach to create event-like sensors for other system could be extremely valuable. It will also be interesting to establish which information is required for which task, i.e., we have shown that the raw histogram data are not necessary for super-resolution, but it may be required for other operations.

## DISCUSSION

The goal of the Pixels2Depth network is to transform the rich information contained within the 4 × 4 histograms to a high-resolution depth map. It is intuitive to think of the network as reformatting the information in the 4 × 4 × 100 histogram array to a 32 × 32 × 1 depth image. The rich information in the temporal domain is used to increase the resolution in the spatial domain. In addition, as we increase in both spatial dimensions, the resulting gain is of order the square root of the initial number of temporal bins, i.e., the two spatial dimensions (*x* and *y*) are both increased by a factor of 8, while the temporal dimension (*z*) is decreased by a factor of 100. If we were to have a different detector with, say, 4096 temporal bins, then we could reasonably expect the gain in each spatial dimension to be of order $\sqrt{4096}=64$ times (*50*). This simple rule of thumb does not take into consideration factors such as the temporal width of the illumination and the impulse response of the detector. However, it can sensibly be used as a guideline for the improvements that can be anticipated.

In this project, we have developed a system that estimates poses of people in 3D from a cost-effective and compact ToF sensor, containing only 4 × 4 pixels. The sensor is small and lightweight, has a low power consumption, and can be easily integrated into consumer electronics such as smartphones or computers. The combined sensor and algorithm is capable of estimating the 3D poses of multiple humans in real time at a maximum range of 3 m and at a frame rate of ≈7 to 8 fps. This frame rate could be significantly improved by leveraging only the core parameters that our explainable framework identifies.

To gain intuition behind why it is possible to recover a high-resolution depth map from a low-resolution depth sensor, we provide an explainable framework to analyze our networks. This framework decomposes the rich and dense information contained within the histograms into the core interpretable features. The output of the linear model provides influence maps that show the impact of each feature on the reconstructions by the networks. This work shows that similar performance to that of Pixels2Depth and Pixels2Pose could be achieved by using only the reduced set of input parameters (the position, amplitude, and widths of the peaks). If new sensors were able to leverage this information, they would be able to provide the relevant data at much higher data rates than are currently possible. Other data types where this is relevant are sonar and radar, where current frame rates of systems can be significantly limited by the requirement to output full datasets (analogous to our full histogram data). Outputting only the core data that are essential for scene reconstruction or object detection could greatly increase the performance of these systems. To learn the core information, the XAI framework that we present in this work could be transferable to other systems. Future work will focus on applying the framework in those cases.

The technology that we present here can be used for action/gesture recognition and will have applications in driver monitoring systems, human-computer interaction, and health care observation. We have detected large-scale objects in this work, and future work will focus on resolving finer features that will open up further applications, e.g., facial structure for face ID applications or finger and hand gestures for sign language identification. The system could also be used for the reconstruction of more general shapes for simultaneous localization and mapping, a navigation technique used by robots and autonomous vehicles.

We note that Pixels2Pose accurately tracks multiple humans in a 3D space, but it does not yet identify specific individuals within a scene. That is to say, Pixels2Pose can track three people simultaneously, but it cannot label each of them separately. This has obvious implications where data protection is an issue. It is not clear yet whether the current sensor would have the resolution in time and space to achieve accurate person identification; however, we note that neural networks have already been used to perform this task on people hidden from view (*62*).

## MATERIALS AND METHODS

Our initial experimental setup for acquiring the training datasets consists of the vl53l5 sensor, mounted on a Raspberry Pi 3B, and a Microsoft Azure Kinect DK camera that records the reference RGB image and the reference depth image. The two sensors, thevl53l5 and Kinect, are placed as close as possible to each other to limit any paralax issues. The radial lens distortion present in the Kinect depth image is corrected for. This ensures that there is a one-to-one correspondence between the spatial locations of the pixels in the depth image and the RGB image. A picture of the setup used for training is shown in the Supplementary Materials.

As the Kinect sensor has a larger field of view than the vl53l5 sensor, we crop the Kinect depth and RGB images appropriately. This means that the data provided to the network for training from the Kinect and vl53l5 sensor have the same field of view. Both the Kinect and vl53l5 sensor operate at about 20 fps; however, the data for both are acquired asynchronously. To match the frames of both devices in time, we save the time at which each frame is recorded and postprocess the data to have as close a match as possible.

Up to three people walk in front of the sensors in random directions, in different positions, and with different arm gestures. We recorded three different datasets containing one, two, or three persons. The one-person dataset contains 7500 frames, and the two- and three-people datasets contain 11,000 frames each. In each case, the first 500 consecutive frames were set aside for validation. A picture of the setup can be found in fig. S1.

## References

[1] M. Zanfir, M. Leordeanu, C. Sminchisescu, The moving pose: An efficient 3D kinematics descriptor for low-latency action recognition and detection, in *Proceedings of the IEEE International Conference on Computer Vision* (IEEE, 2013), pp. 2752–2759.

[2] A. Farooq, A. Jalal, S. Kamal, Dense RGB-D map-based human tracking and activity recognition using skin joints features and self-organizing map. KSII Trans. Internet. Info. Sys. 9, 1856–1869 (2018).

[3] E. Cippitelli, S. Gasparrini, E. Gambi, S. Spinsante, A human activity recognition system using skeleton data from RGBD sensors. Comput. Intell. Neurosci. 2016, 4351435 (2016). 27069469 10.1155/2016/4351435 PMC4812221

[4] A. Mathis, P. Mamidanna, K. M. Cury, T. Abe, V. N. Murthy, M. W. Mathis, M. Bethge, Deeplabcut: Markerless pose estimation of user-defined body parts with deep learning. Nat. Neurosci. 21, 1281–1289 (2018). 30127430 10.1038/s41593-018-0209-y

[5] T. B. Moeslund, A. Hilton, V. Krüger, A survey of advances in vision-based human motion capture and analysis. Comp. Vis. Image Understand. 104, 90–126 (2006).

[6] X. Xiong, W. Min, W.-S. Zheng, P. Liao, H. Yang, S. Wang, S3D-CNN: Skeleton-based 3D consecutive-low-pooling neural network for fall detection. Appl. Intel. 50, 3521–3534 (2020).

[7] Z.-P. Bian, J. Hou, L.-P. Chau, N. Magnenat-Thalmann, Fall detection based on body part tracking using a depth camera. IEEE J. Biomed. Health Inform. 19, 430–439 (2015). 24771601 10.1109/JBHI.2014.2319372

[8] Y. R. Serpa, M. B. Nogueira, Pedro Paulo Macêdo Neto, Maria Andréia Formico Rodrigues, Evaluating pose estimation as a solution to the fall detection problem, in *IEEE 8th International Conference on Serious Games and Applications for Health (SeGAH)* (IEEE, 2020), pp. 1–7.

[9] Q. Wu, G. Xu, F. Wei, L. Chen, S. Zhang, RGB-D videos-based early prediction of infant cerebral palsy via general movements complexity. IEEE Access 9, 42314–42324 (2021).

[10] Y. Gu, S. Pandit, E. Saraee, T. Nordahl, T. Ellis, M. Betke, Home-based physical therapy with an interactive computer vision system, in *2019 IEEE/CVF International Conference on Computer Vision Workshop (ICCVW)*, 2019, pp. 2619–2628.

[11] K. I. Withanage, I. Lee, R. Brinkworth, S. Mackintosh, D. Thewlis, Fall recovery subactivity recognition with rgb-d cameras. IEEE Trans. Industr. Inform. 12, 2312–2320 (2016).

[12] C. Torres, J. C. Fried, K. Rose, B. S. Manjunath, A multiview multimodal system for monitoring patient sleep. IEEE Trans. Multimedia 20, 3057–3068 (2018).

[13] S. Park, J. Y. Chang, H. Jeong, J.-H. Lee, J.-Y. Park, Accurate and efficient 3d human pose estimation algorithm using single depth images for pose analysis in golf, in *Proceedings of the IEEE Conference on Computer Vision and Pattern Recognition Workshops* (IEEE, 2017), pp. 49–57.

[14] B. Lewandowski, J. Liebner, T. Wengefeld, S. Müller, H.-M. Gross, Fast and robust 3D person detector and posture estimator for mobile robotic applications, in *International Conference on Robotics and Automation (ICRA)* (IEEE, 2019), pp. 4869–4875.

[15] Z. Yang, Y. Li, J. Yang, J. Luo, Action recognition with spatio–temporal visual attention on skeleton image sequences. IEEE Trans. Circuits Syst. Vid. Technol. 29, 2405–2415 (2019).

[16] A. S. Keçeli, A. Kaya, A. B. Can, Action recognition with skeletal volume and deep learning, in *25th Signal Processing and Communications Applications Conference (SIU)* (IEEE, 2017), pp. 1–4.

[17] J. Liu, H. Rahmani, N. Akhtar, A. Mian, Learning human pose models from synthesized data for robust RGB-D action recognition. Int. J. Comp. Vis. 127, 1545–1564 (2019).

[18] T. L. Baldi, F. Farina, A. Garulli, A. Giannitrapani, D. Prattichizzo, Upper body pose estimation using wearable inertial sensors and multiplicative Kalman filter. IEEE Sens. J. 20, 492–500 (2020).

[19] X. Yun, E. R. Bachmann, Design, implementation, and experimental results of a quaternion-based Kalman filter for human body motion tracking. IEEE Trans. Robot. 22, 1216–1227 (2006).

[20] T. V. Marcard, R. Henschel, M. J. Black, B. Rosenhahn, G. Pons-Moll, Recovering accurate 3d human pose in the wild using IMUs and a moving camera, in *Proceedings of the European Conference on Computer Vision (ECCV)* (2018), pp. 601–617.

[21] A. Gilbert, M. Trumble, C. Malleson, A. Hilton, J. Collomosse, Fusing visual and inertial sensors with semantics for 3d human pose estimation. Int. J. Comp. Vis. 127, 381–397 (2019).

[22] K. Aminian, B. Najafi, Capturing human motion using body-fixed sensors: Outdoor measurement and clinical applications. Comp. Anim. Virtual Worlds 15, 79–94 (2004).

[23] F. A. de Magalhaes, G. Vannozzi, G. Gatta, S. Fantozzi, Wearable inertial sensors in swimming motion analysis: A systematic review. J. Sports Sci. 33, 732–745 (2015). 25356682 10.1080/02640414.2014.962574

[24] F. Eckardt, A. Münz, K. Witte, Application of a full body inertial measurement system in dressage riding. J. Equine Vet. 34, 1294–1299 (2014).

[25] D. Vlasic, I. Baran, W. Matusik, J. Popović, Articulated mesh animation from multi-view silhouettes, in *ACM SIGGRAPH 2008 Papers* (2008), pp. 1–9.

[26] J. Carranza, C. Theobalt, M. A. Magnor, H.-P. Seidel, Free-viewpoint video of human actors. ACM transactions on graphics (TOG) 22, 569–577 (2003).

[27] K. Iskakov, E. Burkov, V. Lempitsky, Y. Malkov, Learnable triangulation of human pose, in *Proceedings of the IEEE/CVF International Conference on Computer Vision* (IEEE, 2019), pp. 7718–7727.

[28] R. Mehrizi, X. Peng, Z. Tang, X. Xu, D. Metaxas, K. Li, Toward marker-free 3D pose estimation in lifting: A deep multi-view solutio, in *13th IEEE International Conference on automatic Face & Gesture Recognition (FG 2018)* (IEEE, 2018), pp. 485–491.

[29] J. Lee, J. Chai, Paul SA Reitsma, J. K. Hodgins, N. S. Pollard, Interactive control of avatars animated with human motion data, in *Proceedings of the 29th Annual Conference on Computer Graphics and Interactive Techniques* (Association for Computing Machinery, 2002), pp. 491–500.

[30] R. Poppe, Vision-based human motion analysis: An overview. Comp. Vis. Image Understand. 108, 4–18 (2007).

[31] P. C. Bala, B. R. Eisenreich, S. B. M. Yoo, B. Y. Hayden, H. S. Park, J. Zimmermann, Automated markerless pose estimation in freely moving macaques with OpenMonkeyStudio. Nat. Commun. 11, 4560 (2020). 32917899 10.1038/s41467-020-18441-5 PMC7486906

[32] Ł. Kidziński, B. Yang, J. L. Hicks, A. Rajagopal, S. L. Delp, M. H. Schwartz, Deep neural networks enable quantitative movement analysis using single-camera videos. Nat. Commun. 11, 4054 (2020). 32792511 10.1038/s41467-020-17807-z PMC7426855

[33] G. Rogez, P. Weinzaepfel, C. Schmid, LCR-NET++: Multi-person 2D and 3D pose detection in natural images. IEEE Trans. Pattern Anal. Mach. Intell. 42, 1146–1161 (2019). 30640602 10.1109/TPAMI.2019.2892985

[34] D. Mehta, O. Sotnychenko, F. Mueller, W. Xu, M. Elgharib, P. Fua, H.-P. Seidel, H. Rhodin, G. Pons-Moll, C. Theobalt, XNect: Real-time multi-person 3D motion capture with a single RGB camera. ACM Trans. Graph. 39, 82–81 (2020).

[35] A. Benzine, B. Luvison, Q. C. Pham, C. Achard, Single-shot 3D multi-person pose estimation in complex images. Pattern Recog. 112, 107534 (2021).

[36] J. Liu, H. Ding, A. Shahroudy, L.-Y. Duan, X. Jiang, G. Wang, A. C. Kot, Feature boosting network for 3D pose estimation. IEEE Trans. Pattern Anal. Mach. Intell. 42, 494–501 (2020). 30676946 10.1109/TPAMI.2019.2894422

[37] A. Agarwal, B. Triggs, Recovering 3D human pose from monocular images. IEEE Trans. Pattern Anal. Mach. Intell. 28, 44–58 (2006). 16402618 10.1109/TPAMI.2006.21

[38] C.-H. Chen, D. Ramanan, 3D human pose estimation = 2D pose estimation + matching, in *Proceedings of the IEEE Conference on Computer Vision and Pattern Recognition* (2017), pp. 7035–7043.

[39] C. Ionescu, D. Papava, V. Olaru, C. Sminchisescu, Human3.6M: Large scale datasets and predictive methods for 3d human sensing in natural environments. IEEE Trans. Pattern Anal. Mach. Intell. 36, 1325–1339 (2014). 26353306 10.1109/TPAMI.2013.248

[40] D. Mehta, O. Sotnychenko, F. Mueller, W. Xu, S. Sridhar, G. Pons-Moll, C. Theobalt, Single-shot multi-person 3D pose estimation from monocular RGB, in *International Conference on 3D Vision (3DV)* (IEEE, 2018), pp. 120–130.

[41] W. Zhe (2020);https://github.com/wangzheallen/awesome-human-pose-estimation.

[42] Y. Zhou, H. Dong, A. E. Saddik, Learning to estimate 3d human pose from point cloud. IEEE Sens. J. 20, 12334–12342 (2020).

[43] Z. Zhang, L. Hu, X. Deng, S. Xia, Weakly supervised adversarial learning for 3D human pose estimation from point clouds. IEEE Trans. Vis. Comput. Graph. 26, 1851–1859 (2020). 32070974 10.1109/TVCG.2020.2973076

[44] G. Moon, J. Y. Chang, Kyoung Mu Lee., V2V-posenet: Voxel-to-Voxel prediction network for accurate 3D hand and human pose estimation from a single depth map, in *Proceedings of the IEEE Conference on Computer Vision and Pattern Recognition* (CVPR, 2018), pp. 5079–5088.

[45] A. Kirmani, D. Venkatraman, D. Shin, A. Colaço, F. N. C. Wong, J. H. Shapiro, V. K. Goyal, First-photon imaging. Science 343, 58–61 (2014). 24292628 10.1126/science.1246775

[46] L. Chen, H. Wei, J. Ferryman, A survey of human motion analysis using depth imagery. Pattern Recog. Lett. 34, 1995–2006 (2013).

[47] M.-J. Sun, M. P. Edgar, G. M. Gibson, B. Sun, N. Radwell, R. Lamb, M. J. Padgett, Single-pixel three-dimensional imaging with time-based depth resolution. Nat. Commun. 7, 12010 (2016). 27377197 10.1038/ncomms12010 PMC5512623

[48] Z. Zhang, X. Ma, J. Zhong, Single-pixel imaging by means of Fourier spectrum acquisition. Nat. Commun. 6, 6225 (2015). 25649009 10.1038/ncomms7225

[49] Y. Altmann, S. M. Laughlin, M. J. Padgett, V. K. Goyal, A. O. Hero, D. Faccio, Quantum-inspired computational imaging. Science 361, 2298 (2018). 10.1126/science.aat2298 30115781

[50] A. Turpin, G. Musarra, V. Kapitany, F. Tonolini, A. Lyons, I. Starshynov, F. Villa, E. Conca, F. Fioranelli, R. Murray-Smith, D. Faccio, Spatial images from temporal data. Optica 7, 900–905 (2020).

[51] A. Turpin, V. Kapitany, J. Radford, D. Rovelli, K. Mitchell, A. Lyons, I. Starshynov, D. Faccio, 3D imaging from multipath temporal echoes. Phys. Rev. Lett. 126, 174301 (2021). 33988414 10.1103/PhysRevLett.126.174301

[52] M. Zhao, Y. Tian, H. Zhao, M. A. Alsheikh, T. Li, R. Hristov, Z. Kabelac, D. Katabi, A. Torralba, RF-based 3D skeletons, in *Proceedings of the 2018 Conference of the ACM Special Interest Group on Data Communication* (Association for Computing Machinery, 2018), pp. 267–281.

[53] M. Zhao, T. Li, M. A. Alsheikh, Y. Tian, H. Zhao, A. Torralba, D. Katabi, Through-wall human pose estimation using radio signals, in *Proceedings of the IEEE Conference on Computer Vision and Pattern Recognition* (IEEE, 2018), pp. 7356–7365.

[54] M. Nishimura, D. B. Lindell, C. Metzler, G. Wetzstein, Disambiguating monocular depth estimation with a single transient, in *European Conference on Computer Vision* (Springer, 2020), pp. 139–155.

[55] D. B. Lindell, M. O’Toole, G. Wetzstein, Single-photon 3D imaging with deep sensor fusion. ACM Trans. Graph. 37, 113 (2018).

[56] Q. Sun, J. Zhang, X. Dun, B. Ghanem, Y. Peng, W. Heidrich, End-to-end learned, optically coded super-resolution SPAD camera. ACM Transactions on Graphics (TOG) 39, 1–14 (2020).

[57] A. Ruget, S. M. Laughlin, R. K. Henderson, I. Gyongy, A. Halimi, J. Leach, Robust super-resolution depth imaging via a multi-feature fusion deep network. Opt. Express 29, 11917–11937 (2021). 33984963 10.1364/OE.415563

[58] C. Callenberg, Z. Shi, F. Heide, M. B. Hullin, Low-cost SPAD sensing for non-line-of-sight tracking, material classification and depth imaging. ACM Transactions on Graphics (TOG) 40, 1–12 (2021).

[59] Z. Cao, T. Simon, S.-E. Wei, Y. Sheikh, Realtime multi-person 2D pose estimation using part affinity fields, in *Proceedings of the IEEE Conference on computer Vision and pattern Recognition* (IEEE, 2017), pp. 7291–7299.

[60] I. Gyongy, S. W. Hutchings, A. Halimi, M. Tyler, S. Chan, F. Zhu, S. M. Laughlin, R. K. Henderson, J. Leach, High-speed 3D sensing via hybrid-mode imaging and guided upsampling. Optica 7, 1253–1260 (2020).

[61] M. T. Ribeiro, S. Singh, C. Guestrin, “Why should I trust you?" Explaining the predictions of any classifier, in *Proceedings of the 22nd ACM SIGKDD International Conference on Knowledge Discovery and Data Mining* (Association for Computing Machinery, 2016), pp. 1135–1144.

[62] P. Caramazza, A. Boccolini, D. Buschek, M. Hullin, C. F. Higham, R. Henderson, R. Murray-Smith, D. Faccio, Neural network identification of people hidden from view with a single-pixel, single-photon detector. Sci. Rep. 8, 11945 (2018). 30093701 10.1038/s41598-018-30390-0 PMC6085360

