# Supplementary material for: Pixels2Pose: Super-resolution time-of-flight imaging for 3D pose estimation
Source: Sci Adv. 2022 Nov 30;8(48):eade0123. doi: 10.1126/sciadv.ade0123 (PMC9710882; doi:10.1126/sciadv.ade0123)
Supplement: Supplementary file 1 — Materials and Methods Supplementary Text Figs. S1 to S10 Tables S1 to S4 [file sciadv.ade0123_sm.pdf]

Science Advances
AAAS

Supplementary Materials for

Pixels2Pose: Super-resolution time-of-flight imaging for 3D pose estimation

Alice Ruget *et al.*

Corresponding author: Jonathan Leach, j.leach@hw.ac.uk

Sci. Adv. **8**, eade0123 (2022)
DOI: 10.1126/sciadv.ade0123

The PDF file includes:

Materials and Methods
Supplementary Text
Figs. S1 to S10
Tables S1 to S4
Legends for movies S1 to S7

Other Supplementary Material for this manuscript includes the following:

Movies S1 to S7

Supplementary Text

Supplementary material 1: Experimental setup

Fig. S1 shows our experimental setup. The vl53l5 sensor is mounted on a Raspberry Pi 3B. To minimise paralax issues, the Raspberry Pi is placed on top of and close to the Microsoft Azure Kinect DK camera that records the reference depth and RGB images.

Supplementary material 2: Pixels2Pose System

The proposed Pixels2Pose system takes the cropped histogram data of the sensor as the input, i.e. the 4×4 histograms of 100 bins each, and renders the people poses in 3D. Pixels2Pose consists of three steps: (1) a machine learning (ML) network called Pixels2Depth, (2) an ML network called Depth2Pose, and (3) a post-processing module. Pixels2Depth (1) processes the histogram coming from the sensor to render a high-resolution depth map of 32×32 pixels.

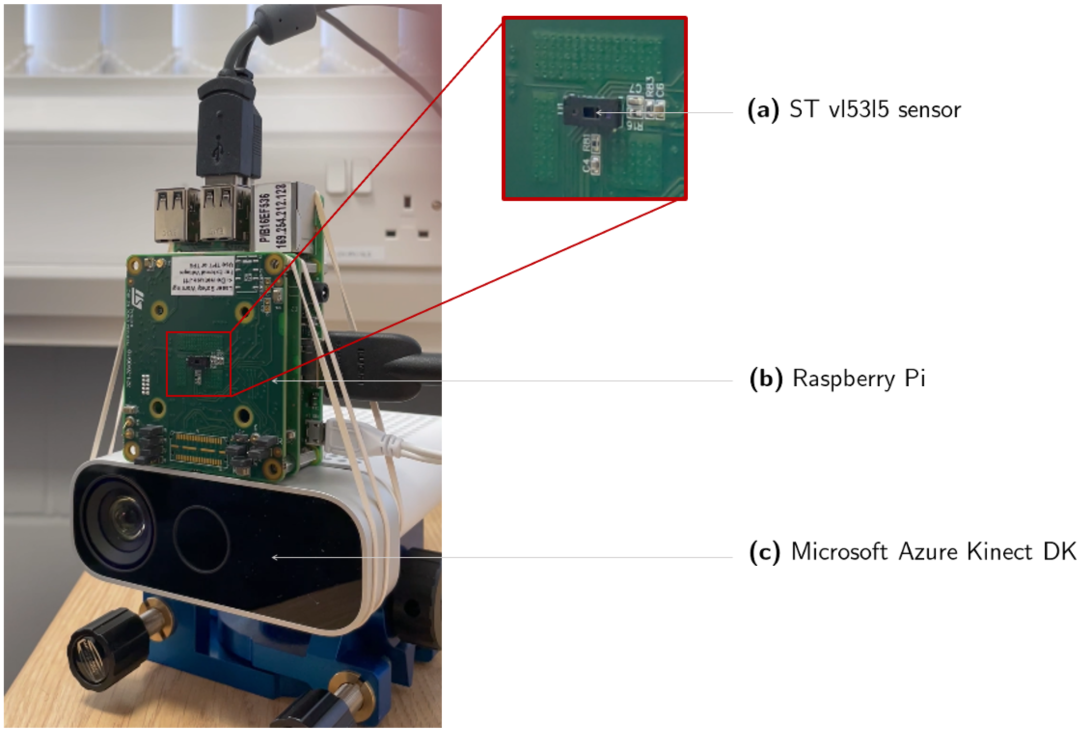

Figure S1: **Photograph of the setup.** The vl53l5 sensor (a) is mounted on a Raspberry Pi 3B (b). The Raspberry Pi is placed on top of a Microsoft Azure Kinect DK camera (c) that records the reference depth and RGB images.

Depth2Pose (2) processes this 32×32 depth map to output the 2D position of joints and limbs of all people present. Finally, module (3) recovers the skeleton in 3D from the output of both Pixels2Depth and Depth2Pose.

I. Pixels2Depth

I.1 Architecture

Pixels2Depth takes as input the raw 4×4×100 histograms and outputs a 32×32 depth image. It consists of a series of 3D convolutional operations that progressively up-sample the initial histogram data in the transverse spatial dimensions and down-sample in the time/depth dimension. ReLu function is used as an activation function in the convolution layers. The architecture of

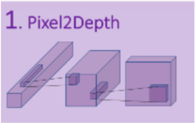

operators	number of filters	filter's shape	strides	output shape
**Input : 4x4x100 histograms recorded by vl53l5 sensor**				
conv3d	64	(3,3,3)	(1,1,1)	(4,4,100,64)
conv3d	64	(3,3,3)	(1,1,1)	(4,4,100,64)
conv3d	64	(3,3,3)	(1,1,1)	(4,4,100,64)
maxpool3d	-	-	(1,1,2)	(4,4,50,64)
deconv3d	32	(3,3,3)	(2,2,1)	(8,8,50,32)
conv3d	32	(3,3,3)	(1,1,1)	(8,8,50,32)
conv3d	32	(3,3,3)	(1,1,1)	(8,8,50,32)
maxpool3d	-	-	(1,1,2)	(8,8,25)
deconv3d	16	(3,3,3)	(2,2,1)	(16,16,25,16)
conv3d	16	(3,3,3)	(1,1,1)	(16,16,25,16)
conv3d	16	(3,3,3)	(1,1,1)	(16,16,25,16)
maxpool3d	-	-	(1,1,2)	(16,16,13,16)
deconv3d	8	(3,3,3)	(2,2,1)	(32,32,13,8)
conv3d	8	(3,3,3)	(1,1,1)	(32,32,13,8)
conv3d	8	(3,3,3)	(1,1,1)	(32,32,13,8)
maxpool3d	-	-	(1,1,2)	(32,32,7,8)
Reshape	-	-	-	(32,32,56)
conv2d	1	(3,3)	(1,1)	(32,32)
**Output : 32x32 depth image**				

Figure S2: **Pixels2Pose architecture.** Pixels2Depth processes the raw $4\times4\times100$ histograms through a series of 3D convolutional layers to render a 32×32 depth image. ReLu function is used as an activation function in the convolution layers.

the network is shown in Fig. S2.

I.2 Training

The network is trained on captured data from the ToF sensor and images from the Kinect camera. The inputs are the $4\times4\times100$ histograms recorded by the sensor. The label depth images are recorded simultaneously by an Azure Kinect camera. We down-sample the depth images recorded by the Kinect to 32×32 pixels with bicubic interpolation.

We implemented the network using the Keras API for Tensorflow. The network was trained separately for the case of one, two, or three people. The number of training images is respectively 7000, 9 500 and 9 500. We trained on an NVIDIA RTX 6000 GPU for 2000 epochs. We minimize the $l2$ loss between the predicted and the label depth images and use the Adam optimizer. During the training, a batch-mode learning method with a batch size of M = 32 was used, and the loss was defined as

$$\mathcal{L}(\theta) = \frac{1}{MN}\sum_{m=1}^{M}\sum_{n=1}^{N}\left(D_{m,n}(\theta) - \widehat{D}_{m,n}\right)^2, \quad (1)$$

with M the number of images within one batch, N the number of pixels of each image, θ the trainable parameters of the network, D the predicted depth , $\widehat{D}$ the ground truth depth.

II. Depth2Pose

Depth2Pose processes the 32×32 depth images previously estimated with Pixels2Pose to render the 2D poses of people.

II.1 Pose estimates

The 2D pose estimates consist of the position of key-point anatomical body parts and of the limbs that connect them. The anatomical parts estimated are the following: nose, neck, eyes, ears, shoulders, wrists, elbows, knees, hips, ankles, and the limbs estimated connect these parts such as Left shoulder – Left elbow, right knee – right ankle, etc. More precisely, the pose estimates consist of a set of 18 2D confidence maps $S = (S_1, S_2, ..., S_{18})$ of the position of each body part and of a set of vectors of part affinities fields (PAFs), which corresponds to the degree of association between two parts (54). The set of part affinity fields $L = (L_1, L_2, ..., L_{19})$ has 19 vector fields, where each L_i is two 32×32 images per limbs.

II.2 Architecture of Depth2Pose

Our model takes as input the 32×32 depth images and outputs the sets of 2D pose estimates S and L. The architecture of our network is inspired by the vision-based network OpenPose (54). The architecture is shown in Fig. S3. It consists of three stages and each stage is made up of two branches: branch 1 predicts the set S of confidence maps, branch 2 predicts the set L of PAFs. At each stage, predictions from the previous stage are concatenated and used as input of the next stage's branches. Branches 1 and 2 have similar structures described in Fig. S3. The contracting path consists of the application of one 3×3 convolution, followed by a rectified linear unit and a 2×2 max-pooling operation, this is repeated three times. The expansive path consists of three convolutional layers with a stride of 2 for up-sampling.

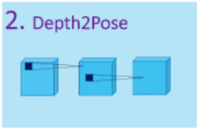

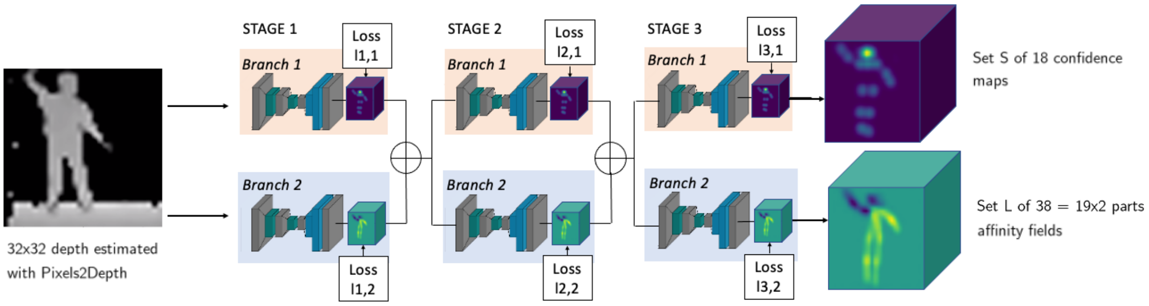

Branch 1

operators	number of filters	filter's shape	strides	output shape
**Input:** $32 \times 32 \times C_{stage}$ with $C_1 = 1, C_2 = 18+38 = 56, C_3 = 56$				
conv2d	100	(3,3)	(1,1)	(32,32,100)
maxpool2d	-	-	(2,2)	(16,16,100)
conv2d	100	(3,3)	(1,1)	(16,16,100)
maxpool2d	-	-	(2,2)	(8,8,100)
conv2d	100	(3,3)	(1,1)	(8,8,100)
conv2dTranspose	100	(3,3)	(2,2)	(16,16,100)
conv2dTranspose	100	(3,3)	(2,2)	(32,32,100)
conv2d	18	(3,3)	(2,2)	(32,32,18)
**Output : set S of 18 confidence maps**				

Branch 2

operators	number of filters	filter's shape	strides	output shape
**Input:** $32 \times 32 \times C_{stage}$ with $C_1 = 1, C_2 = 18+38 = 56, C_3 = 56$				
conv2d	100	(3,3)	(1,1)	(32,32,100)
maxpool2d	-	-	(2,2)	(16,16,100)
conv2d	100	(3,3)	(1,1)	(16,16,100)
maxpool2d	-	-	(2,2)	(8,8,100)
conv2d	100	(3,3)	(1,1)	(8,8,100)
conv2dTranspose	100	(3,3)	(2,2)	(16,16,100)
conv2dTranspose	100	(3,3)	(2,2)	(32,32,100)
conv2d	38	(3,3)	(2,2)	(32,32,38)
**Output : set L of 38 = 19x2 part affinity fields**				

Figure S3: **Depth2Pose architecture.** Depth2Pose processes 32×32 depth maps and renders 2D pose estimates. The 2D pose estimates consist of confidence maps of the position of key body parts, and part affinity fields corresponding to the degree of association between them. The network consists of 3 stages, each stage containing 2 branches, one predicting confidence maps, and one predicting the PAFs. After each stage, the predictions from the two branches are concatenated for the next stage.

II.3 Training

The network is trained on experimentally captured data. The input depth image is recorded using an Azure Kinect camera. The ground truth 2D pose estimates S and L are obtained using the network OpenPose (*54*) applied to an RGB image, captured simultaneously by the Kinect. The loss $l_{s,1}$ at the sth stage of branch 1 is defined as

$$l_{s,1}(\theta) = \frac{1}{MN} \sum_{m=1}^{M} \sum_{n=1}^{N} \sum_{i=1}^{18} \left(S_i^{s,m,n}(\theta) - \widehat{S}_i^{s,m,n} \right)^2 , \tag{2}$$

with M the number of images within one batch, N the number of pixels of each image, θ the trainable parameters of the network, S_i^s the confidence map predicted at stage s for the body part i, $\widehat{S}_i^s$, the ground truth confidence map for body part i. The loss $\mathcal{L}_{s,2}$ for branch 2 is defined as

$$l_{s,2}(\theta) = \frac{1}{MN} \sum_{m=1}^{M} \sum_{n=1}^{N} \sum_{i=1}^{19} \left(L_i^{s,m,n}(\theta) - \widehat{L}_i^{s,m,n} \right)^2 , \tag{3}$$

with L_i^s the part affinity field predicted at stage s for limb i, and $\widehat{L}_i^s$ the ground truth part affinity field for limb i. The training loss function is the sum of the losses of each stage and is defined as

$$\mathcal{L}(\theta) = \sum_{s=1}^{3} l_{s,1}(\theta) + l_{s,2}(\theta) \tag{4}$$

We implemented the network again using the Keras API for Tensorflow. Depth2Pose was trained separately for the case of one, two, or three people. The number of training images is respectively 7000, 9500 and 9500. We trained on an NVIDIA RTX 6000 GPU for 2000 epochs.

III. Post-processing module

This module renders the pose in 3D by combining the outputs of Pixels2Depth and Depth2Pose. Depth2Pose gives the location in 2D of each body part. The module reports the 2D location onto the depth map estimated by Pixels2Depth to find the depth coordinate.

In some cases, the estimates of the pose and the depth map do not perfectly overlap, leading to outliers in the depth of certain body parts. Outliers in depth values can also arise when occlusions happen (either self-occlusion or multi-person occlusion), as it will be the front-most depth value read for the occluded joints.

We correct for the outliers in depth by using the following criterion:

$$z_c = \begin{cases} m & \text{if } |z - m| > 50\text{cm} \\ z & \text{else,} \end{cases} \tag{5}$$

with z_c the depth after correction, z the initial estimated depth, m the median of the depths of all the body parts. Fig. S4 shows results before and after applying the correction module. Note finally that we take into account the magnification at different distances and convert the data to an orthographic projection. This ensures that humans that are far away from the sensor and therefore appear smaller in the initial 3D reconstruction are scaled appropriately and appear the correct size in the final images.

Supplementary material 3: Evaluation of the performance of Pixels2Depth

We evaluate the performance of the depth upsampling network Pixels2depth using several errors: the root mean squared error, the mean absolute difference (MAD) and the delta metrics defined as

$$MAD = \frac{1}{N}\sum_{i=1}^{N}(|d_i - \widehat{d_i}|), \tag{6}$$

$$RMSE = \sqrt{\frac{1}{N}\sum_{i=1}^{N}(d_i - \widehat{d_i})^2}, \tag{7}$$

$$\text{delta metric :}\% \text{ of } d_i \text{ s.t. } |d_i - \widehat{d_i}| < \delta, \tag{8}$$

with N the total number of pixels, δ is an error threshold, d_i the estimated depth value and $\widehat{d_i}$ the reference value.

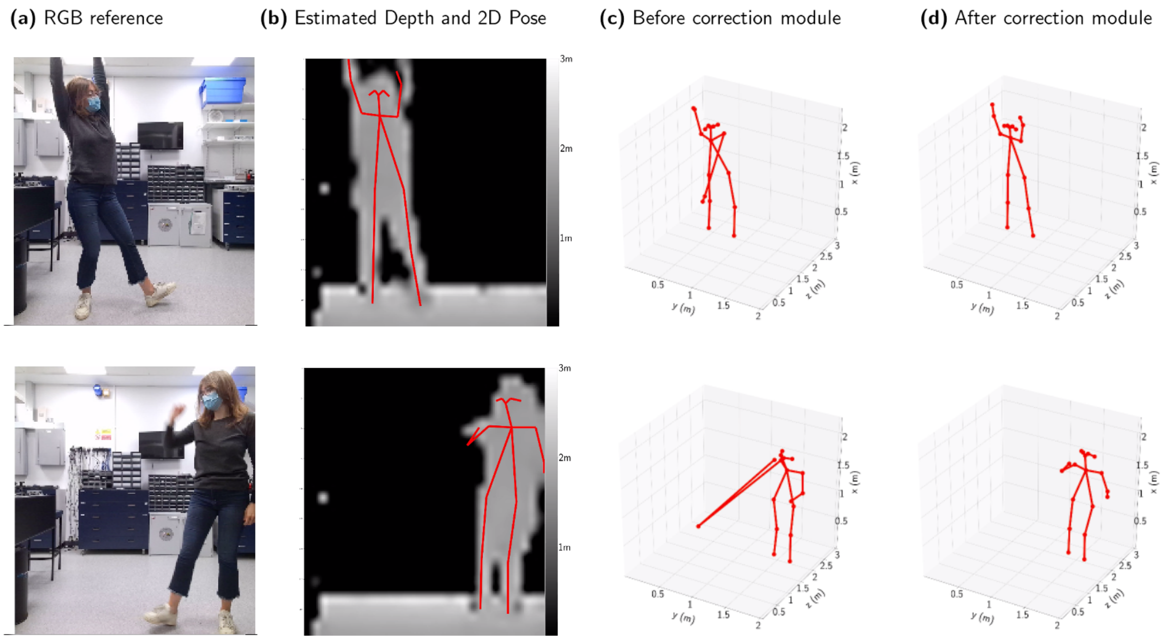

Figure S4: **Results before and after the correction module.** (a) shows the reference RGB image taken by the Kinect. (b) shows the superposition of the depth map estimates of Pixels2Depth and the 2D pose estimate of Depth2Pose. (c)/(d) show the results in 3D before/after applying the correction module.

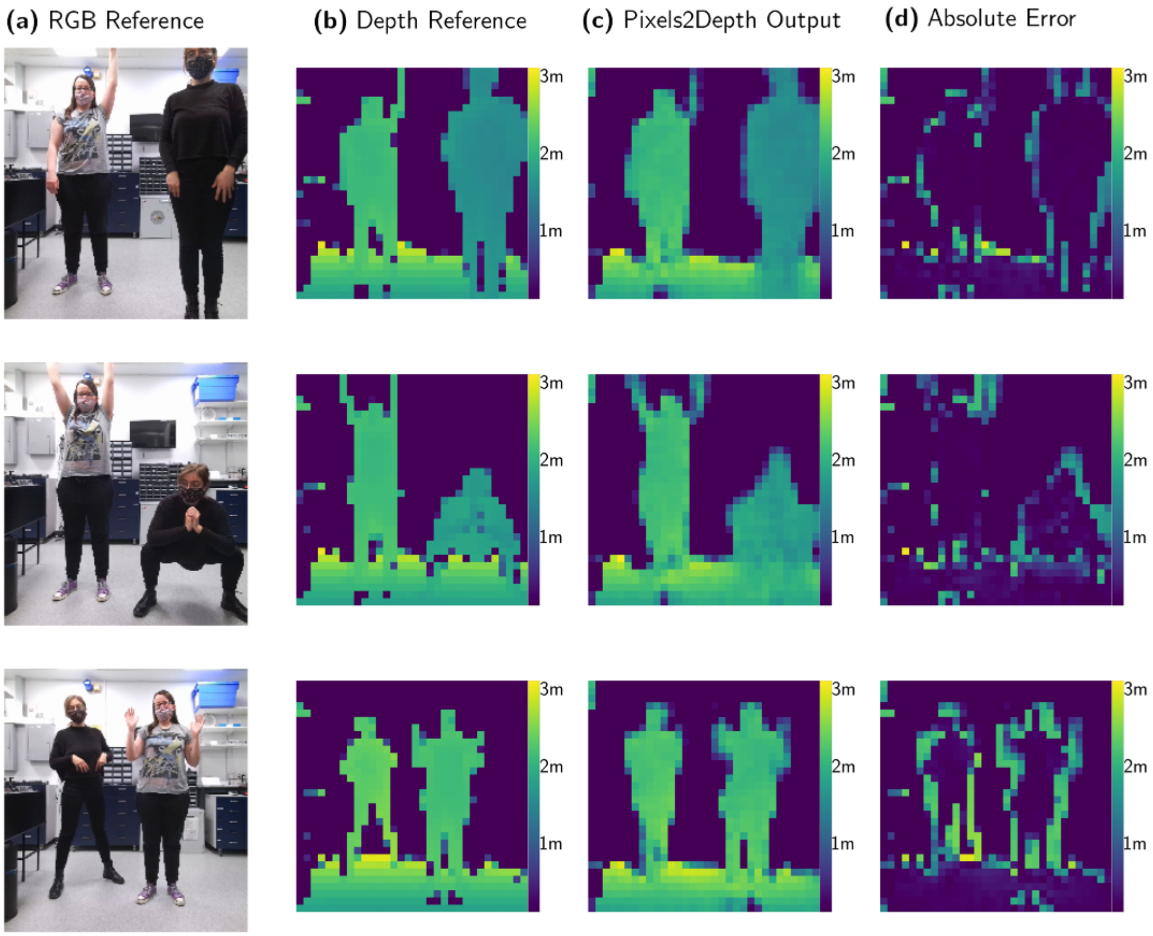

Figure S5: **Evaluation of Pixels2Depth network.** (a) shows the reference RGB image taken by the Kinect, (b) shows the depth reference from the Kinect down-sampled to 32x32 pixels, (c) show the reconstructed depth from Pixels2Depth, and (d) shows the absolute error between (b) and (c).

scenario	$RMSE$ (cm)	MAD (cm)	$\delta = 5$ cm (%)	$\delta = 10$ cm (%)	$\delta = 15$ cm (%)
1 person	49.1	17	78	84	86
2 people	47.4	18.1	70	79	83
3 people	49.0	19.5	63	74	79

Table S1: **Evaluation of the performance of Pixels2Depth.** We evaluate against ground truth data in terms of typical depth metrics, such as RMSE, MAD and delta metrics, evaluated on the dense depth map.

We evaluate Pixels2Depth on 500 validation images of each scenario of one, two and three people. The results are shown in Table S1. The MAD is of order 20 cm and the RMSE is order 50 cm. We note that the large RMSE is attributed to large differences between the edges of the reconstructed depth images and the ground truths, as shown in Figure S5. However, this error is not carried through to the pose estimation metrics, which perform much better as they are not subject to edge effects.

Supplementary material 4: Reducing the memory footprint

The model Pixels2Depth consists of 368 929 parameters of type float32 and takes about 4.7 MB of memory. The model Depth2Pose consists of 2 517 768 parameters which requires 30 MB. To optimize the memory requirements of the models, we use the Tensorflow lite converter. This converts a saved Tensorflow model into a Tensorflow lite model, by applying post-training quantization to the trainable weights from floating-point to integer, which has 8-bits of precision. After this conversion, Pixels2Depth requires only 740KB and Depth2Pose 4.9MB. We can directly test these lite models on a Raspberry Pi 4, in real time together with the acquisition of the data. We achieve a frame rate of 1 fps for both the acquisition and the processing of the data.

We evaluate the performance of the lite models on the 1500 images of the validation dataset. Table S2 and Table S3 show the performance of the lite model in red and the original model in black. We calculate, for each joint, the root mean squared error between the estimated and

	$RMSE_x$ (cm)	$RMSE_y$ (cm)	$RMSE_z$ (cm)	AE(cm)
neck	5.9 / 5.4	6.5 / 6.0	9.3 / 8.5	9.8 / 9.5
shoulders	6.3 / 5.8	12.5 / 12.4	9.9 / 9.2	12.5 / 12.3
hips	4.5 / 4.4	9.0 / 8.8	9.1 / 9.1	10.2 / 10.2
knees	5.8 / 5.6	11.4 / 11.1	10.1 / 10.1	11.9 / 11.9
ankles	8.3 / 7.9	15.4 / 15.1	11.8 / 11.3	15.4 / 15.1
elbows	17.5 / 17.7	20 / 19.9	13.8 / 13.4	19.7 / 19.6
wrists	22.6 / 22.6	25.7 / 26	17.2 / 17.6	25.7 / 25.9

Table S2: **Evaluation of the performance of the lite models (red) and the original model using root mean squared error metrics.** The lite model is of size 5 MB, the original model is of size 35MB. We report the root mean squared error between the estimated and the ground truth position of each joint for each axis x,y, and z.

	PCK-15 (%)	PCK-20 (%)	PCK-30 (%)	% detected
neck	62 / 80.0	87.0 / 88.0	91.0 / 92.0	100 / 100
shoulders	72.2 / 72.5	79.9 / 80.2	85.3 / 86.3	99.7 / 99.5
hips	77.1 / 77.8	83.3 / 83.3	90.6 / 91.6	99.5 / 100
knees	72.5 / 72.1	81.1 / 81.7	88.6 / 89.8	98.1 / 99.8
ankles	61.1 / 62.1	73.3 / 74.4	85.1 / 86.4	96.1 / 98.4
elbows	61 / 60.9	68 / 68.6	74.9 / 75.4	95.1 / 96
wrists	48.9 / 50	56.6 / 57.5	64.6 / 65.1	88.9 / 84.9

Table S3: **Evaluation of the performance of the lite models (red) and the original model using percentages of correct key points PCK.** The lite model is of size 5 MB, the original model is of size 35MB. We report the percentages of correct key points (PCK-15, PCK-20, PCK-30), i.e the ratio of estimated body parts for which the distance to the ground truth is below 15, 20, and 30 cm respectively. We also report the percentage of detected parts.

the ground truth positions along the x, y, and z (depth) axes, the average error AE and the percentages of correct key points (PCK-15, PCK-20, PCK-30), i.e the ratio of estimated body parts for which the distance to the ground truth is below 15, 20 and 30 cm, respectively. The converted lite models achieve similar performance as the original model.

Supplementary material 5: Test on other objects

We tested Pixels2Pose on scenes containing other objects, e.g., chairs and a water container. In these cases, no human skeleton can be retrieved. The intermediate outputs of the networks

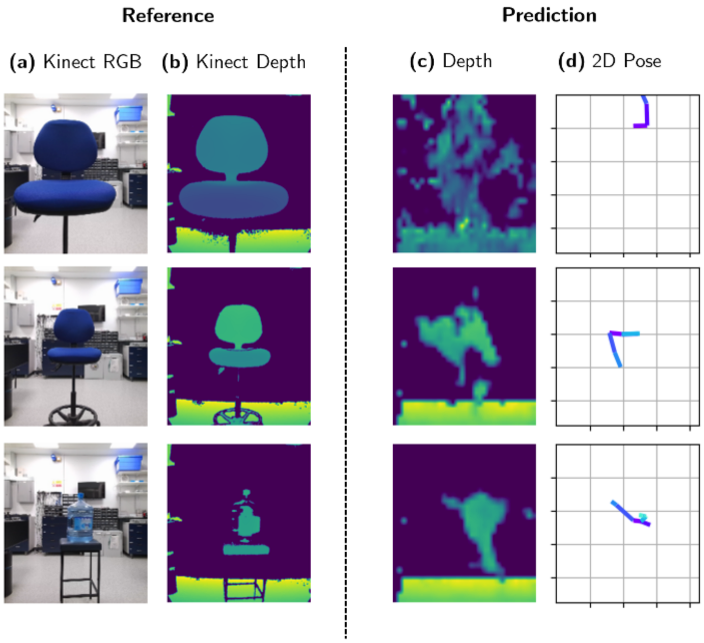

Figure S6: **Results for non-human shapes.** We test Pixels2Pose on scenes containing chairs (first and second row) and a water container (third row). (a) and (b) show the reference RGB and depth images acquired by a Kinect camera. (c) shows the output of Pixel2Depth and (d) the output of Depth2Pose. Only some arms and shoulders are misidentified. To differentiate human shapes from other objects, a threshold of a minimum of five limbs can be set. Pixels2Pose can identify those objects as not humans.

Pixel2Depth and Depth2Pose are shown in Figure S6. We see that some arms and shoulders are misidentified. To differentiate human shapes from other objects, a threshold of a minimum of five limbs can be set.

Supplementary material 6: Failure cases

Fig. S7 shows examples of the most common failure cases of Pixels2Pose. The network could fail to identify arm movements when multiple people are present in the scene, e.g. in the case of three people present, arms can be misplaced alongside the body, as in Fig. S7 (a). Moreover, movements over multiple time frames are sometimes unrealistic, e.g. changes in the position of arms and legs that are too rapid are occasionally observed, as in Fig. S7 (a). We also observe

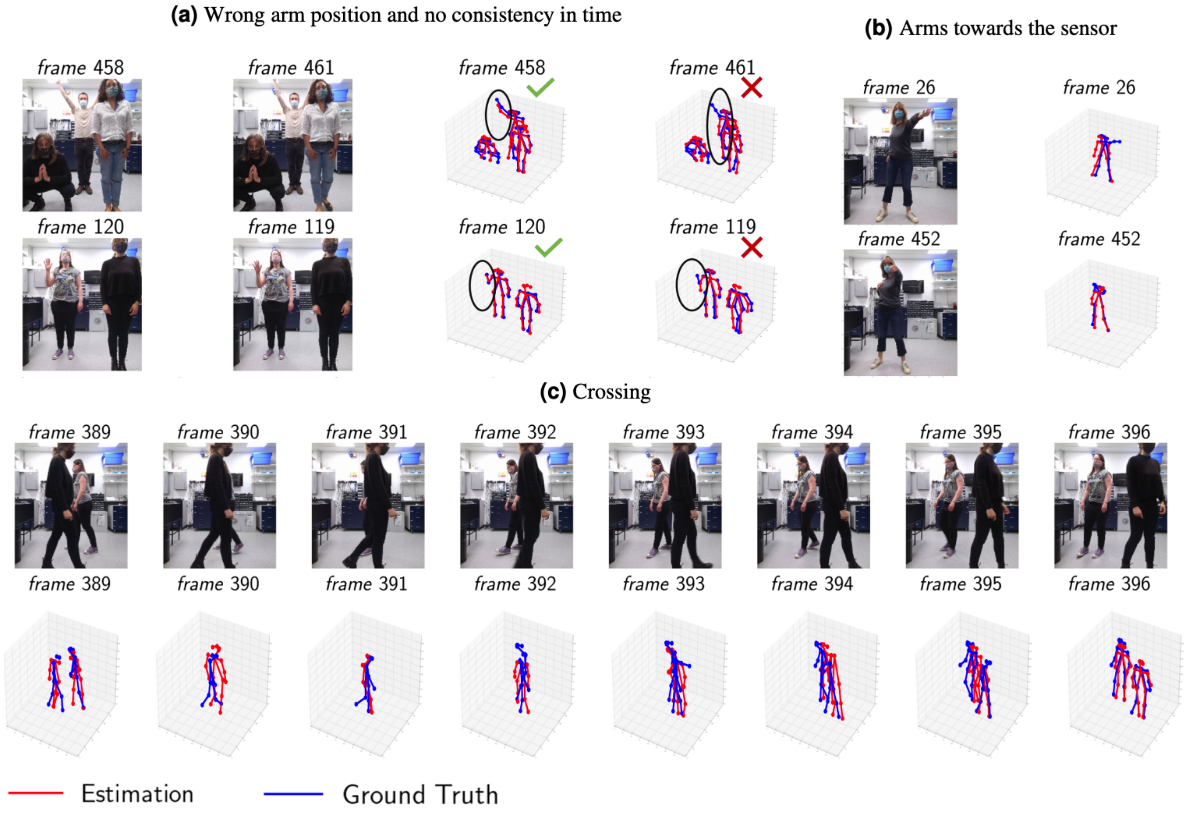

Figure S7: **Examples of failure cases.** (a) represents the case wrong arm position. (b) shows cases when arms were positioned in the axis of the sensor. (c) shows the issue when people are crossing.

that people can disappear from the frame when crossing behind one another, as in Fig. S7 (c). The system might fail to identify arms that are directed towards the sensor as in Fig. S7 (b).

Supplementary material 7: Explanation model

We provide here the details of the linear model used to gain intuition on the proposed network.

I. Pixels2Depth

The explanation model for Pixels2Depth is a linear function A that outputs 32×32 depth map d from a set x of n features. In the one person scenario, there are 48 features: the amplitude,

width and position of the main peak for each of the 16 pixels. In the two person scenario, there are 96 features because two peaks for each pixel are taken into account.

We solve the following optimization problem:

$$min_A\big(1/(2*n_{samples})||W(d-xA)||_2^2+\alpha||A||_1\big), \tag{9}$$

with $n_{samples} = 2000$ the number of perturbed samples around the instance of interest, $x \in \mathbb{R}^{n_{samples}\times n}$ the Gaussian parameters, $d \in \mathbb{R}^{n_{samples}\times 1024}$ the depth map predicted by Pixels2Depth, $A \in \mathbb{R}^{n\times 1024}$ the weights of the linear surrogate model, $W \in \mathbb{R}^{n_{samples}\times n_{samples}}$ a diagonal matrix representing the proximity weights of the sampled instances to the instance of interest, $\alpha = 0.0001$ the regularization parameter.

The perturbed instances are generated by changing the amplitude, width and position of the original peaks for a random combination of pixels by up to 10%. The proximity weights are defined as the inverse of the sum of all the percentages applied to the parameters.

The linear model gives access directly to the weights applied to each parameter of the histogram to build the final depth map. Figures S8, S9 and S10 show the absolute weights applied to all the amplitudes, widths and positions of the peaks to build the final depth map. We observe the weights are spatially consistent, i.e. the amplitudes of the top pixels have more impact on the top of the final depth map.

II. Pixels2Pose

The explanation model for PixelsPose is a linear function A that outputs the set p of 3D coordinates for fourteen body parts (neck, nose, shoulders, elbows, wrists, knees, hips,feet) of each person present from the set x of n Gaussian features. In the one person scenario, p is of length $n_p = 42$ (3×14) ; in the two person scenario, p is of length 84 since two people are taken into account.

We solve the following optimization problem:

$$min_A\big(1/(2*n_{samples})||W(p-xA)||_2^2+\alpha||A||_1\big), \tag{10}$$

with $x \in \mathbb{R}^{n_{samples}\times n}$ the set of Gaussian parameters, $p \in \mathbb{R}^{n_{samples}\times n_p}$ the 3D coordinates of the body parts, $A \in \mathbb{R}^{n\times n_p}$ the weights of the linear surrogate model, $W \in \mathbb{R}^{n_{samples}\times n_{samples}}$ diagonal matrix representing the proximity weights of the sampled instances to the instance of interest, $\alpha = 0.01$ the regularization parameter.

Supplementary material 8: Performance in other environments

To demonstrate that the trained Pixels2Pose network is transferable between different environments, we took new data with the vl53l5 sensor in a new room and from two different angles. We evaluate the accuracy of the estimated 3D poses on 500 images taken in two different environments. We report in Table S4 the error in position in x,y and z and the percentages of correct key points (PCK-15, PCK-20, PCK-30), i.e. the ratio of estimated body parts for which the distance to the ground truth is below 15, 20, and 30 cm respectively. The data shows that the Pixels2Pose network recovers the 3D pose in an environment in which it was not trained, thus demonstrating the versatility of our system.

	$RMSE_{x/y/z}$ (cm)	AE(cm)	PCK-15 (%)	PCK-20 (%)	PCK-30 (%)	% detected
neck	8.2 / 8.8 / 11.9	11.3	82.0	92.0	93.0	100
shoulders	9.1 / 13 / 10	12.2	75.2	87.5	89.8	99.5
hips	32.5 / 21.1 / 13.8	28.4	45.2	58.9	68.8	100
knees	20.4 / 44.7 / 10	42.7	17.5	27.3	36.1	99.8
ankles	33.9 / 49.8 / 17.3	56.1	7	13.1	23.1	98.4
elbows	16.6 / 19.3 / 7.1	19.8	52.2	66.2	76.4	96
wrists	25.5 / 30.5 / 8.2	31.1	23.4	40.9	60.8	84.9

Table S4: **Evaluation of the performance in other environment.** We report the root mean squared error between the estimated and the ground truth position of each joint for each axis x,y, and z. We also report the percentages of correct key points (PCK-15, PCK-20, PCK-30), i.e. the ratio of estimated body parts for which the distance to the ground truth is below 15, 20, and 30 cm respectively. We also report the percentage of detected parts.

Supplementary videos

Supplementary video 1 : Validation results with one person

Supplementary video 2 : Validation results with two people

Supplementary video 3 : Validation results with three people

Supplementary video 4 : Pixels2Pose estimates with reference for one person

Supplementary video 5 : Pixels2Pose estimates with reference for two people

Supplementary video 6 : Pixels2Pose estimates with reference for three people

Supplementary video 7 : Results in other environments not used for training

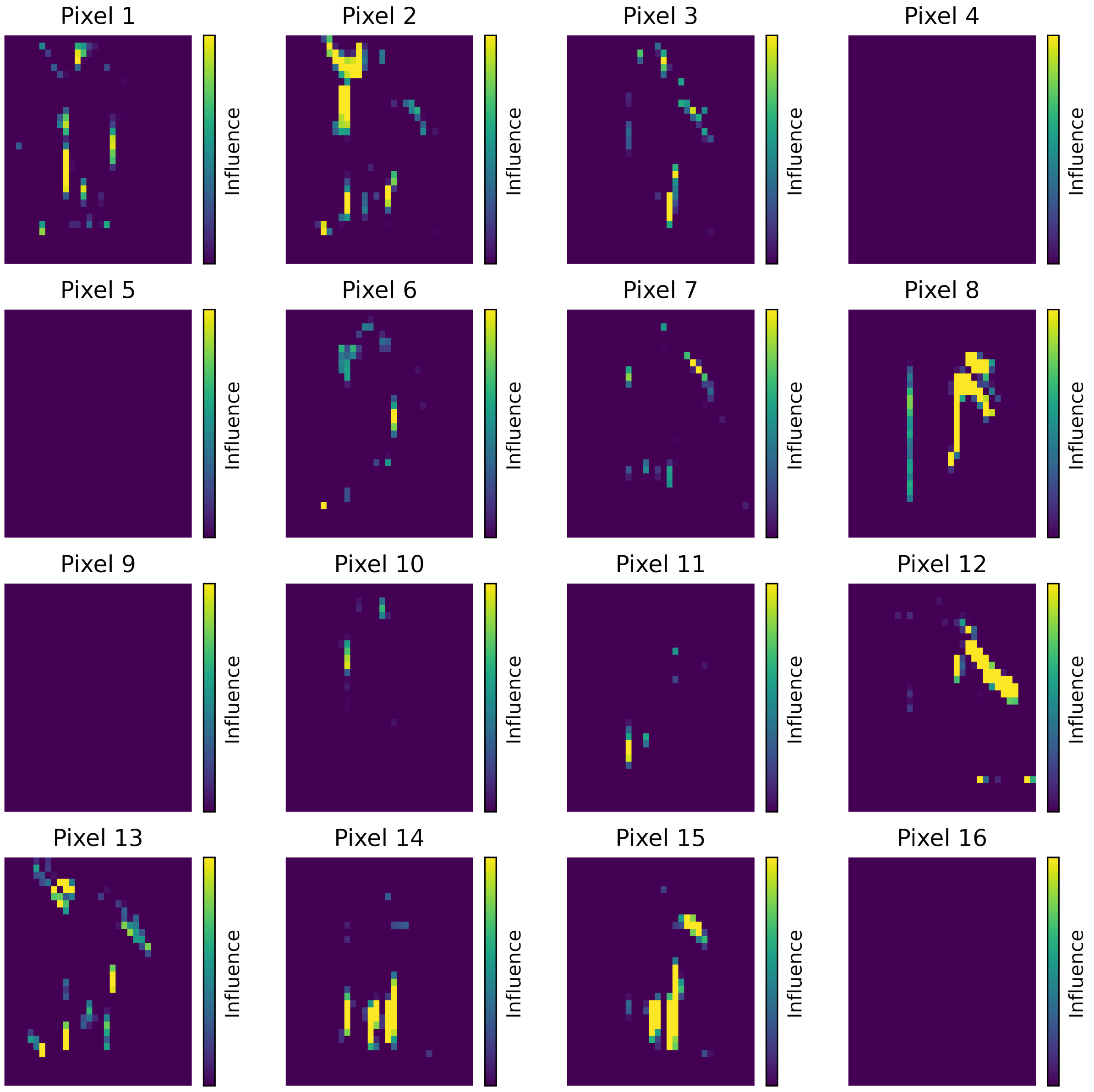

Figure S8: **Weights of the explanation model for Pixels2Depth applied to the sixteen amplitudes of the histogram data.** Each subplot #i represents the weights applied to the amplitude of pixel #i in the sensor's histogram input data.

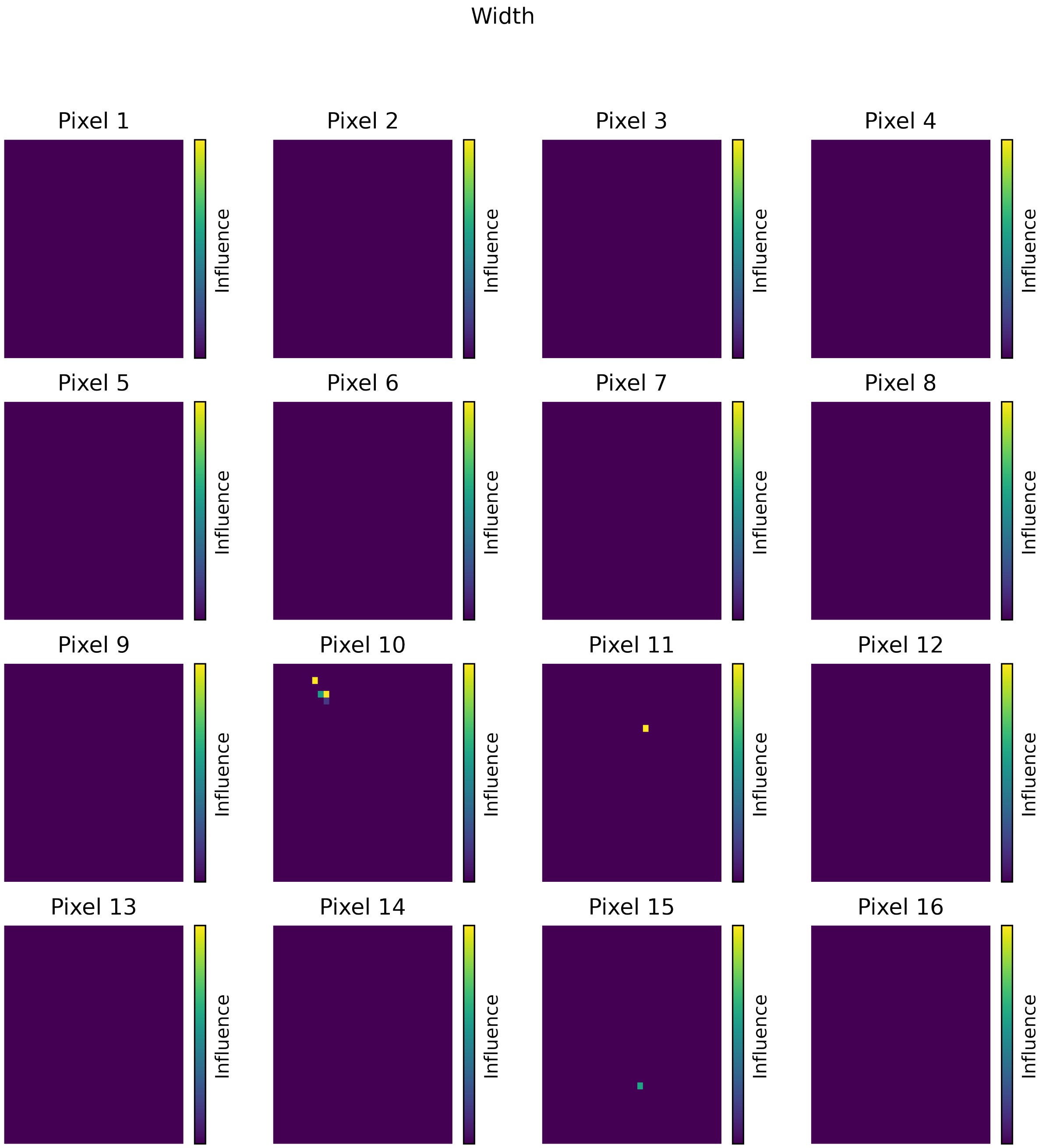

Figure S9: **Weights of the explanation model for Pixels2Depth applied to the sixteen widths of the histogram data.** Each subplot #i represents the weights applied to the width of pixel #i in the sensor's histogram input data.

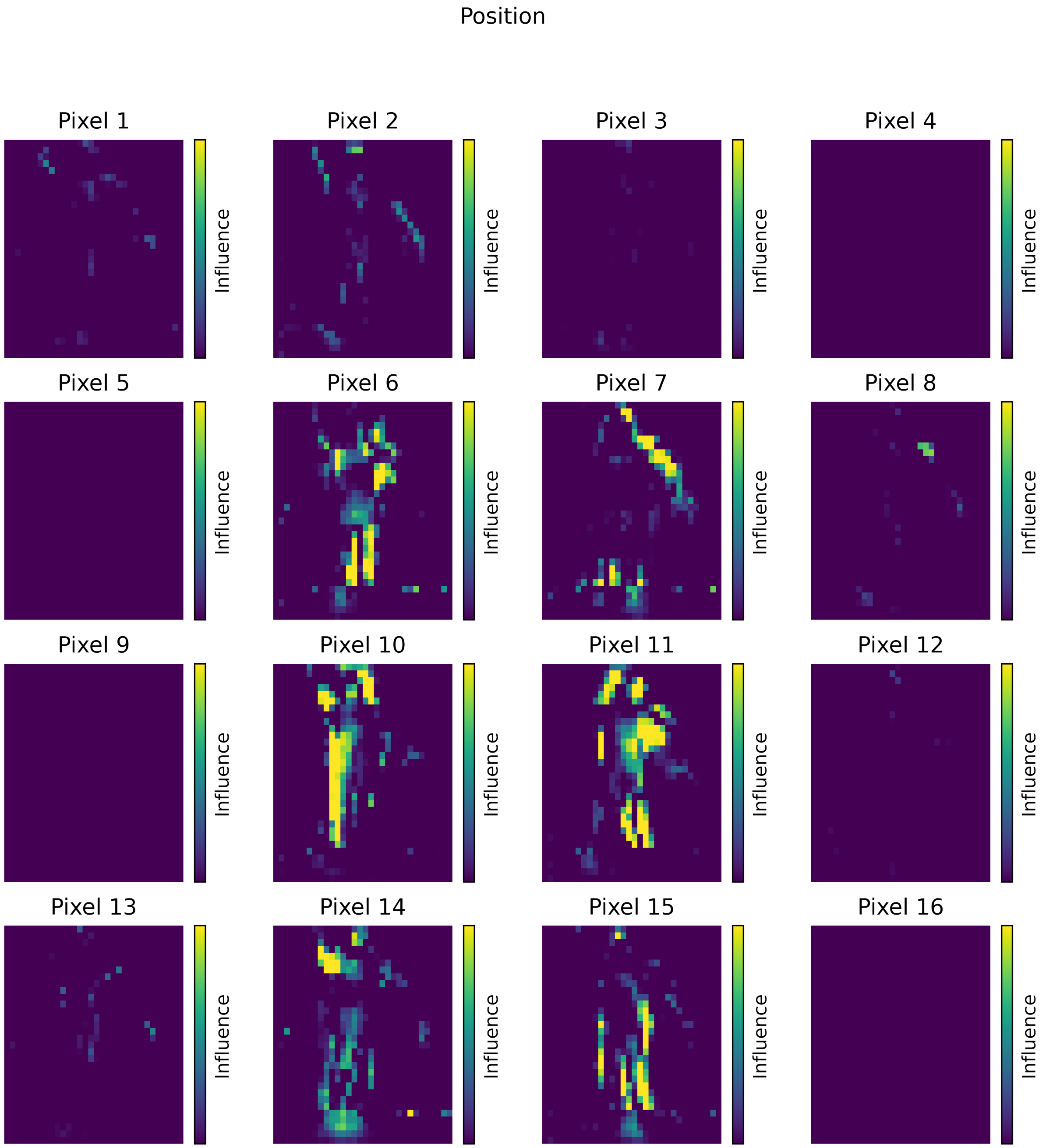

Figure S10: **Weights of the explanation model for Pixels2Depth applied to the sixteen positions of the histogram data.** Each subplot #i represents the 32 weights applied to the positions of pixel #i in the sensor's histogram input data.
